# Efficacy of traditional Chinese exercises on cognitive function in older adults: a systematic review and meta-analysis of randomised controlled trials

**DOI:** 10.1093/ageing/afag168

**Published:** 2026-06-08

**Authors:** Bin Li, Lirong Yu, Na Li, Kaisy Xinhong Ye, Jiuyu Guo, Luwen Cao, Jiatong Shan, Yecheng Li, Xiu Wang, Tih-Shih Lee, Brian K Kennedy, John Suckling, Andrea Maier, Wenbin Wu, Lei Feng

**Affiliations:** Department of Geriatrics, Hospital of Chengdu University of Traditional Chinese Medicine, No. 39 Shi-er-qiao Road, Chengdu 610072, Sichuan, China; School of Nursing, Shandong Second Medical University, Weifang, Shandong, China; Laboratory of Molecular Pharmacology, Jilin Provincial Key Laboratory of Biomacromolecules of Chinese Medicine, Jilin Ginseng Academy, Changchun University of Chinese Medicine, Changchun, Jilin, China; Healthy Longevity Translational Research Program, Yong Loo Lin School of Medicine, National University of Singapore, Singapore, Singapore; Healthy Longevity Translational Research Program, Yong Loo Lin School of Medicine, National University of Singapore, Singapore, Singapore; Centre for Healthy Longevity, @AgeSingapore, National University Health System, Singapore, Singapore; Healthy Longevity Translational Research Program, Yong Loo Lin School of Medicine, National University of Singapore, Singapore, Singapore; Healthy Longevity Translational Research Program, Yong Loo Lin School of Medicine, National University of Singapore, Singapore, Singapore; Centre for Healthy Longevity, @AgeSingapore, National University Health System, Singapore, Singapore; Department of Psychological Medicine, Yong Loo Lin School of Medicine, National University of Singapore, Singapore, Singapore; College of Chemistry, Jilin University, Changchun, China; Department of Neurology, Beijing Chuiyangliu Hospital, Beijing, China; Neuroscience and Behavioural Disorders Programme, Duke-NUS Medical School, Neuroscience & Behavioural Disorders Programme, Singapore, Singapore; Healthy Longevity Translational Research Program, Yong Loo Lin School of Medicine, National University of Singapore, Singapore, Singapore; Centre for Healthy Longevity, @AgeSingapore, National University Health System, Singapore, Singapore; Department of Biochemistry, Yong Loo Lin School of Medicine, National University of Singapore, Singapore, Singapore; Department of Psychiatry, University of Cambridge, Herchel Smith Building for Brain and Mind Sciences, Cambridge, UK; Healthy Longevity Translational Research Program, Yong Loo Lin School of Medicine, National University of Singapore, Singapore, Singapore; Department of Human Movement Sciences, Faculty of Behavioral and Movement Sciences, Amsterdam, Netherlands; Department of Geriatrics, Hospital of Chengdu University of Traditional Chinese Medicine, No. 39 Shi-er-qiao Road, Chengdu 610072, Sichuan, China; Centre for Healthy Longevity, @AgeSingapore, National University Health System, Singapore, Singapore; Department of Psychological Medicine, Yong Loo Lin School of Medicine, National University of Singapore, Singapore, Singapore

**Keywords:** cognitive function, older adults, traditional Chinese exercises, meta-analysis, randomised controlled trial, systematic review

## Abstract

**Background:**

Cognitive impairment is a significant health concern among older adults, highlighting the need for non-pharmacological interventions, such as mind–body exercises. However, a comprehensive synthesis of the effects of various traditional Chinese exercises (TCEs) on cognitive function in older adults is lacking.

**Methods:**

A systematic review and meta-analysis of randomised controlled trials (RCTs) was conducted. Six databases were searched from inception to 2 May 2024 for studies examining the effects of TCEs on cognitive outcomes in adults aged 60 years and older. Studies were included if they were RCTs involving TCEs and reported outcome measures for global cognition, or individual cognitive domains.

**Results:**

Twenty-eight RCTs with a total of 2297 participants were included. Meta-analysis revealed that TCEs led to significant improvements in global cognition: Montreal Cognitive Assessment [mean differences (MD) = 1.67; 95% confidence interval (CI): (1.20, 2.14)], Mini-Mental State Examination [MD = 0.76; 95% CI: (0.04, 1.48)]; executive function: Trail Making Test (B-A) [MD = −7.96; 95% CI: (−15.34, −0.59)], Category Fluency for Animals [MD = 2.96, 95% CI (2.08, 3.85)]; working memory: Digit Span-Backwards [MD = 0.48; 95% CI: (0.07, 0.90)]; processing speed: Digit Symbol Coding [MD = 4.16; 95% CI: (1.82, 6.50)]; memory function: Memory Quotient [MD = 13.13; 95% CI: (4.06, 22.20)], Auditory Verbal Learning Test: immediate recall [MD = 1.13; 95% CI: (0.07, 2.20)], short-term delayed recognition [MD = 0.80; 95% CI: (0.28, 1.32)] and long-term delayed recognition [MD = 1.38; 95% CI: (0.68, 2.09)].

**Conclusions:**

TCEs are effective in improving cognitive function in older adults, particularly in domains such as global cognition, executive function, working memory, processing speed and memory function. However, given the methodological limitations and heterogeneity of the included studies, these findings require confirmation in further large-scale, high-quality RCTs.

## Introduction

Cognitive ability is crucial for older adults to maintain functional independence, including managing daily activities, finances and medications. Population ageing poses a major global health and economic challenge: by 2050, the number of individuals over 60 years is expected to reach 2.1 billion [1], with those aged 80 years or older tripling to 426 million. Cognitive decline, driven by neuronal dysfunction and loss, is a common consequence of ageing [2]. The prevalence of dementia is projected to rise from 55 million in 2019 to 139 million in 2050 [3], with associated global costs increasing from US$1.3 trillion in 2019 to an estimated $2.8 trillion by 2030 [4].

Alzheimer’s disease (AD), the most common form of dementia, is a neurodegenerative disease characterised by cognitive dysfunctions and memory impairment [5, 6]. Mild cognitive impairment (MCI), a transitional stage between normal cognition and dementia, progresses to dementia in 30%–40% of cases within 5 years [7, 8]. Dementia poses a significant public health burden on older adults, with high costs for medical and informal caregiving [9]. Increasing evidence suggests that lifestyle factors can influence the risk of dementia and MCI. The 2024 Lancet Commissions report indicated that 45% of dementia cases could be potentially prevented or delayed by addressing modifiable risk factors, with physical inactivity as a key contributor [10]. The FINGER trial underscored the importance of exercise in dementia prevention [11], and a recent network meta-analysis confirmed the benefits of exercise across multiple cognitive domains, influenced by frequency, intensity, duration, type, volume or total intervention length and progression [12]. Emerging evidence suggests that mind–body exercise, including traditional Chinese exercises (TCEs), may hold potential for ameliorating neurodegenerative disorders.

TCEs, originated in China about 3000 years ago, are multimodal mind–body exercises that combine moderate movements, breathing exercises, social interaction and meditation [13], making them particularly suitable for older adults who may struggle with high-intensity fitness regimens. Various forms of TCEs, including Tai Chi [14], Baduanjin (Eight Brocades of Qigong) [15], Yijinjing (Muscle Tendon Strengthening Classic) [16], Wuqinxi (Five Animals Qigong Practice) [17], Liuzijue (The Six Healing Sounds) [18], traditionally used to prevent and manage cognitive decline. Evidence supports cognitive benefits for Tai Chi in older adults with type 2 diabetes (T2D) and MCI [19], and for Baduanjin in enhancing cognitive ability and reducing physical frailty in cognitive frailty [20]. A bibliometric analysis [21] highlights growing research on TCEs for neurodegenerative diseases.

Despite this increasing interest, a comprehensive review of cognitive effects of various TCEs is lacking. Therefore, this systematic review and meta-analysis of RCTs aims to address this gap in older adults.

## Methods

This study was registered in PROSPERO with reference number #CRD 42024539587 and reported according to the Preferred Reporting Items for Systematic Reviews and Meta-Analysis (PRISMA) checklist [22].

### Search strategy

A systematic search was conducted in PubMed, Embase, PsycINFO, Cochrane Central Register of Controlled Trials, Web of Science and Scopus from database inception to 2 May 2024, using a combination of Medical Subject Headings (MeSH) terms and free-text terms related to participant characteristics, interventions and outcomes. The complete search strategies are provided in Appendix S1.

Following deduplication, two reviewers independently screened titles and abstracts, and subsequently reviewed full-text articles to determine eligibility. Discrepancies were resolved through discussion or, when necessary, adjudication by a senior reviewer.

### Selection criteria

Studies were included if they met the following criteria: (i) participant: adults aged 60 years and older; (ii) intervention: at least one arm of the study involved a TCE intervention; (iii) comparison: control group participants engaged in either an active (non-TCE activities such as fitness training, simple handicrafts, health education) or passive (e.g. usual care, waitlist) comparison; (iv) outcomes: reported at least one measure of cognitive function (e.g. global cognition, executive function, working memory); (v) study design: RCT.

Studies were excluded if they met any of the following criteria: (i) not published in English or the full-text was unavailable; (ii) the intervention combined TCEs with other active interventions; (iii) the control group received another form of TCEs; (iv) duplicate reporting of the same study; (v) publication type was a review, editorial, conference abstract, commentary or protocol; (vi) studies that reported only neuroimaging, physiological or biomarker outcomes without including any standardised cognitive function scale tests.

### Data extraction

Data were extracted independently by two authors using a structured data extraction form. The form was developed based on previous review experience and was refined iteratively during the initial extraction phase to ensure clarity and consistency. Any discrepancies between extractors were resolved through discussion or with the input of a third reviewer. The extracted data included:

General study information: author names, year of publication, country/location of study. Participant demographics: health condition, sample size, mean age, sex. Health condition was categorised according to the classifications reported in the original studies.

Intervention characteristics: number of study arms, type(s) of TCEs, frequency and duration of the intervention, mode of instruction, characteristics of the control group and cognitive outcome measures. Data for cognitive outcomes were extracted at the time points reported by the primary studies. To ensure comparability, the primary analysis focused on the first post-intervention time point, defined as the assessment conducted immediately after the final intervention session.

### Risk of bias assessment

Two independent reviewers assessed the risk of bias in each included study using the Cochrane Collaboration’s tool for assessing the risk of bias in randomised trials. This tool evaluates bias across six domains: selection bias, performance bias, detection bias, attrition bias and reporting bias and other potential sources of bias. Each domain was rated as low risk, unclear risk and high risk. Discrepancies between reviewers were resolved through consensus with the broader research team.

### Statistical analysis and data synthesis

Meta-analyses were carried out only for outcomes measured by common, standardised tests that were used by at least two independent studies. All meta-analyses in this study were conducted using the Review Manager (RevMan 5.4), with respective meta-regressions performed in STATA 17.0 [23]. Given the multiple cognitive domains and analyses, we did not apply formal multiplicity corrections. Instead, we pre-specified Montreal Cognitive Assessment (MoCA) as the primary outcome, defined a limited set of key secondary outcomes, and treated the remaining analyses as exploratory, interpreting them cautiously. An alpha level of 0.05 was used for all hypothesis testing. Between-study heterogeneity was quantified by *I*^2^ statistic, with values of 25%, 50% and 75% indicating low, medium and high heterogeneity, respectively. Mean differences (MDs) and standard deviations (SDs) were calculated alone with their corresponding 95% confidence intervals (CIs).

To assess the robustness of findings, subgroup analyses were carried out based on different characteristics of the control group. The strategy for subgroup analyses was pre-specified in our PROSPERO protocol, which stated: ‘Additional subgroup analyses will be considered depending on the retrieved data.’ We also performed sensitivity analyses based on a leave-one-out methods.

We used a random-effects model to pool the effect of traditional exercise on cognitive function, given anticipated clinical and methodological heterogeneity across studies. To explore potential sources of heterogeneity, we pre-specified meta-regression analyses for outcomes with at least 10 studies, examining moderators including age, sex, health status, type of TCE, instruction mode, control group type, intervention frequency and duration.

### Publication bias

Publication bias was visually assessed using funnel plots generated in RevMan 5.4. Egger’s regression tests [24] were performed in STATA 17.0 for outcomes with 10 or more studies to formally test for asymmetry. A *P* value <.05 was considered statistically significant, indicating evidence of publication bias.

## Results

### Study selection

**Figure 1:**
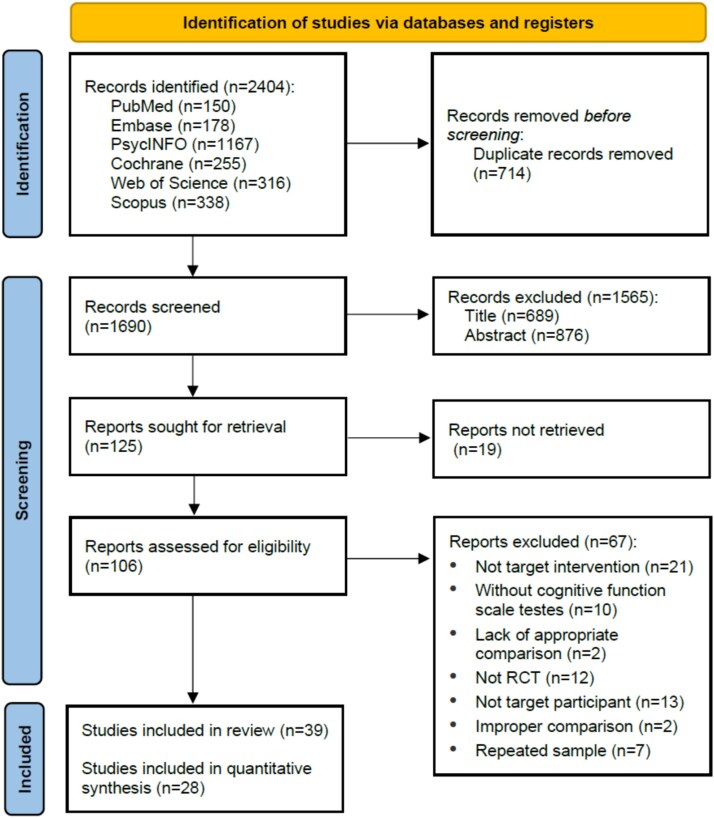
PRISMA flow diagram of screening and selection process.

A total of 2404 records were identified through the database searches (Figure 1). After removing duplicates (*n* = 714), 1690 articles were screened by titles and abstract. Of these, 125 articles were considered potentially eligible, and full-text were retrieved for 106 of these articles. Following full-text review, 28 RCTs (*n* = 2297) met the inclusion criteria and were included in the meta-analysis.

### Characteristics of included studies

The characteristics of the included studies are summarised in Appendix S2.

#### Study setting and participant characteristics

Studies were conducted in China (*n* = 20), USA (*n* = 7) or Thailand (*n* = 1). Participant diagnoses varied, with MCI (*n* = 11), non-dementia with MoCA>22 or Mini-Mental State Examination (MMSE)>24 (*n* = 6), cognitive frailty (*n* = 2), Parkinson’s disease (*n* = 2), and one study each for mild dementia, geriatric depression, heart failure, cognitive impairment and osteoarthritic knee, T2D with MCI, post-stroke cognitive impairment, and multiple chronic conditions without dementia.

#### Intervention characteristics

The most frequently studied TCEs were Tai Chi (18 studies, 1793 participants), Baduanjin (7 studies, 391 participants), other forms including Wuqinxi, Liuzijue (Six Healing Sounds) and Wu Xing Ping Heng Gong (3 studies, 113 participants). Instruction modes included on-site teaching by coach (25 studies), online guidance (1), exercise video (1), not mentioned (1), control group conditions were categorised as active group (16) and care-as-usual group (12).

#### Training load

Session duration varied: 60 minutes was most common (16 studies), followed by 120 (2), 50 (3), 40 (2), 30 (2) and 20 minutes (3). Frequency was typically 3 times/week (17 studies), with other schedules including 7, 5, 4, 2, 1 or 1–2 times/week. Intervention duration ranged from 10 to 52 weeks, most frequently 24 weeks (10 studies) or 12 weeks (7 studies). Detailed parameters for each study are summarised in Table S2.

#### Outcome measures

Commonly reported cognitive domains and assessment tools included: global cognition: MoCA (*n* = 13), MMSE (*n* = 7); executive function: Trail Making Test (TMT) (*n* = 9), Category Fluency for Animals (*n* = 3); working memory: Digit Span-Backwards (DS-B) (*n* = 5); processing speed: Digit Symbol Coding (DSC) (*n* = 3); attention: Digit Span-Forwards (DS-F) (*n* = 4); visuospatial ability: Clock Drawing Task (CDT) (*n* = 2); memory function: Memory Quotient (MQ) (*n* = 4), Auditory Verbal Learning Test (AVLT) (*n* = 6).

### Risk of bias in included studies

The risk of bias across domains is summarised in Appendix S3. (i) Random sequence generation (7.1% high risk, 39.3% unclear, 53.6% low risk), (ii) allocation concealment (10.7% high risk, 17.9% unclear, 71.4% low risk), (iii) blinding of participants and personnel (100% high risk, 0% unclear, 0% low risk), (iv) blinding of outcome assessment (0% high risk, 64.3% unclear, 35.7% low risk), (v) incomplete outcome data (10.7% high risk, 10.7% unclear, 78.6% low risk), (vi) selective reporting (14.3% high risk, 10.7% unclear, 75% low risk) and (vii) other bias (28.6% high risk, 46.4% unclear, 25% low risk).

### Effectiveness on cognitive function

#### Global cognition

**Figure 2:**
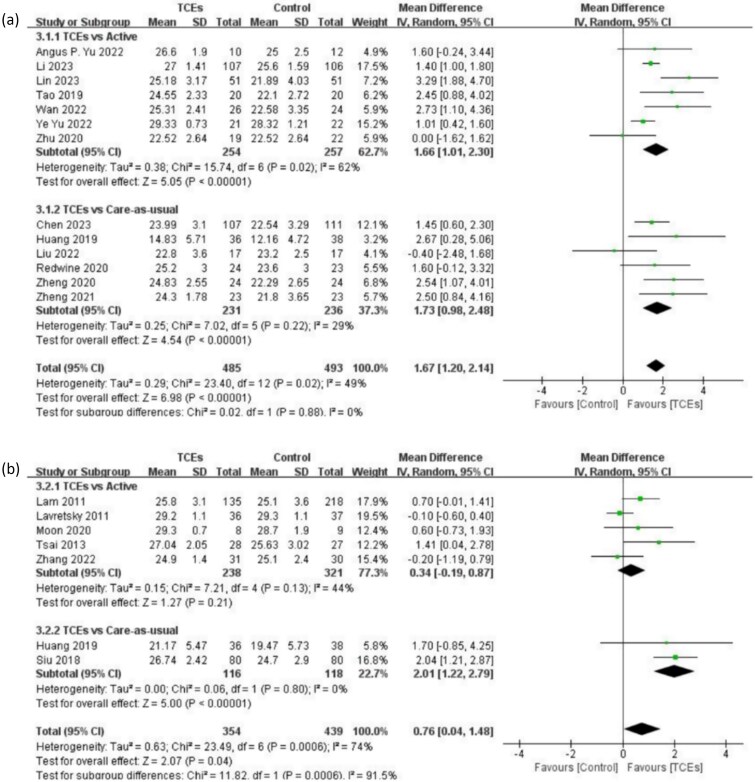
Forest plot of global cognition. (a) Montreal Cognitive Assessment (MoCA); (b) Mini-Mental State Examination (MMSE).

Thirteen RCTs (*n* = 978) reported outcomes for the MoCA [25]. Meta-analysis revealed a significant positive effect of TCEs on global cognition [MD = 1.67; 95% CI: (1.20, 2.14); *P* < .00001; Figure 2a]. Heterogeneity between studies was classified medium (*P* = .02; *I^2^* = 49%). Subgroup analyses based on the type of TCE and the characteristics of the control group showed that TCEs were associated with greater improvements in MoCA scores compared to both active control [MD = 1.66, 95% CI (1.01, 2.30); *P* < .00001] and care-as-usual group [MD = 1.73, 95% CI (0.98, 2.48); *P* < .00001].

Seven RCTs (*n* = 793) reported outcomes for the MMSE [26]. Meta-analysis identified a significant positive effect of TCEs on global cognition [MD = 0.76; 95% CI: (0.04, 1.48); *P* = .04; Figure 2b] with high heterogeneity (*P* = .0006; *I^2^* = 74%). In subgroup analyses, a significant difference was noted when the control group was care-as-usual [MD = 2.01, 95% CI (1.22, 2.79); *P* < .00001], but not when the control group was active [MD = 0.34, 95% CI (−0.19, 0.87); *P* = .21].

#### Executive function

**Figure 3:**
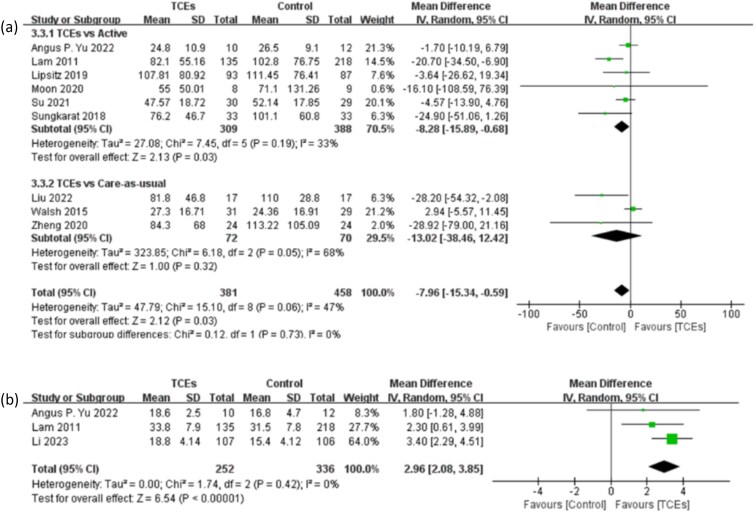
Forest plot of executive function. (a) Trail Making Test (TMT) (b-a); (b) Category Fluency for Animals.

Nine RCTs (*n* = 839) assessed the effects of TCEs on executive function using the TMT. TMT Part B minus Part A (B-A) was used to evaluate task-switching ability, a subdomain of executive function [27]. Smaller difference scores indicate better switching ability. Meta-analysis showed that TCEs were associated with a significant decrease in TMT (B-A) scores [MD = −7.96; 95% CI: (−15.34, −0.59); *P* = .03; Figure 3a], indicating improved task-switching ability compared to the control group. Heterogeneity between studies was medium (*P* = .06; *I^2^* = 47%). Subgroup analyses based on the characteristics of the control group revealed a significant difference between the TCE intervention group and the active control group [MD = −8.28; 95% CI: (−15.89, −0.68); *P* = .03], but not between the TCE intervention group and the care-as-usual control group [MD = −13.02; 95% CI: (−38.46, 12.42); *P* = .32].

Three RCTs (*n* = 588) evaluated the effect of Tai Chi on executive function using the Category Fluency for Animals [28]. Meta-analysis showed a significant improvement in Category Fluency for Animals in the Tai Chi group compared to the control group [MD = 2.96; 95% CI (2.08, 3.85); *P* < .00001; Figure 3b] with no heterogeneity between studies (*P* = .42; *I^2^* = 0%).

#### Working memory

**Figure 4:**
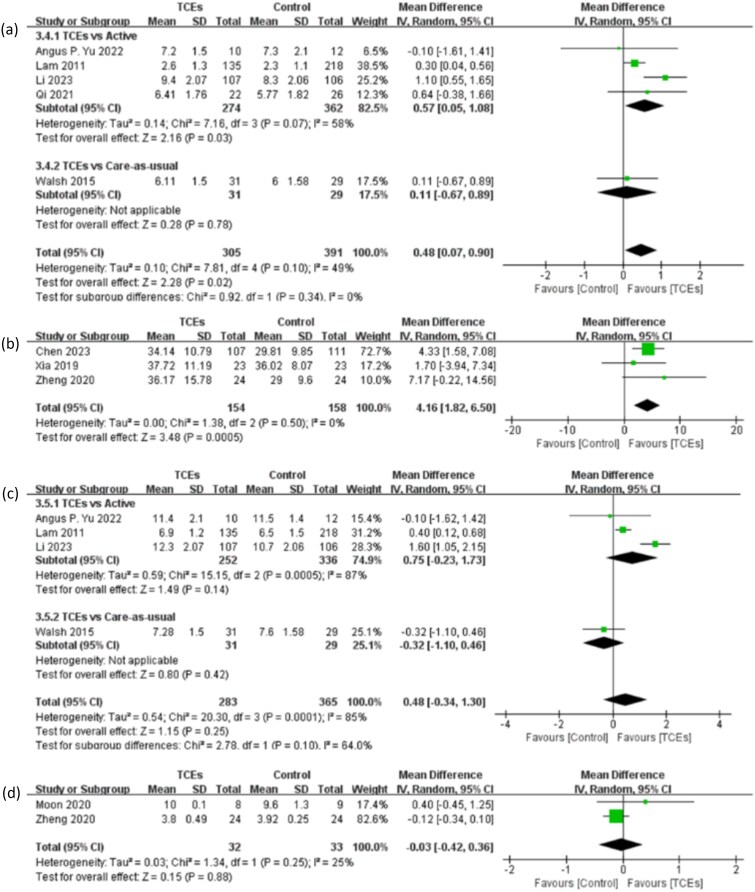
Forest plot of working memory, processing speed, attention and visuospatial ability. (a) Digit Span-Backwards (DS-B); (b) Digit Symbol Coding (DSC); (c) Digit Span-Forwards (DS-F); (d) Clock Drawing Task (CDT).

Five RCTs (*n* = 696) reported the effects of TCEs training on working memory using DS-B. DS-B was used to measure working memory by repeating the sequence verbally in the reverse order (backward) of numerical digits [29]. The pooled analysis of DS-B indicated a significant benefit of TCEs on working memory [MD = 0.48; 95% CI: (0.07, 0.90); *P* = .02; Figure 4a] with medium heterogeneity between studies (*P* = .10; *I^2^* = 49%). Subgroup analyses showed a significant difference between the TCE group and the active control group [MD = 0.57; 95% CI: (0.05, 1.08); *P* = .007], but not between the TCE group and the care-as-usual control group [MD = 0.11; 95% CI: (−0.67, 0.89); *P* = .78].

#### Processing speed

Three RCTs (*n* = 302) presented outcomes for processing speed using the DSC, in which participant was required to convert numbers into corresponding symbols as soon as possible in 90 s [30]. Meta-analysis showed that the TCE group had faster processing speed than the control group [MD = 4.16; 95% CI: (1.82, 6.50); *P* = .0005; Figure 4b] with no heterogeneity among studies (*P* = .50; *I^2^* = 0%).

#### Attention

Four RCTs (*n* = 648) assessed the effects of TCEs training on attention via the DS-F. DS-B was used to measure attention by repeating the sequence verbally in the same order (forward) of numerical digits [29]. The pooled analysis revealed no significant difference between the TCE group and the control group [MD = 0.48; 95% CI: (−0.34, 1.30); *P* = .25; Figure 4c], and high heterogeneity was detected between studies (*P* = .0001; *I^2^* = 85%). Subgroup analyses showed no significant differences between the TCE group and either the active control group [MD = 0.75; 95% CI: (−0.23, 1.73); *P* = .14] or the care-as-usual control group [MD = −0.32; 95% CI: (−1.10, 0.46); *P* = .42].

#### Visuospatial ability

Two RCTs (*n* = 65) assessed the effects of TCE training on visuospatial ability using CDT [31]. Pooled analysis indicated no significant difference between the TCE group and the control group [MD = −0.03; 95% CI: (−0.42, 0.36); *P* = .88; Figure 4d]. The level of heterogeneity between studies was low (*P* = .25; *I^2^* = 25%).

#### Memory function

**Figure 5:**
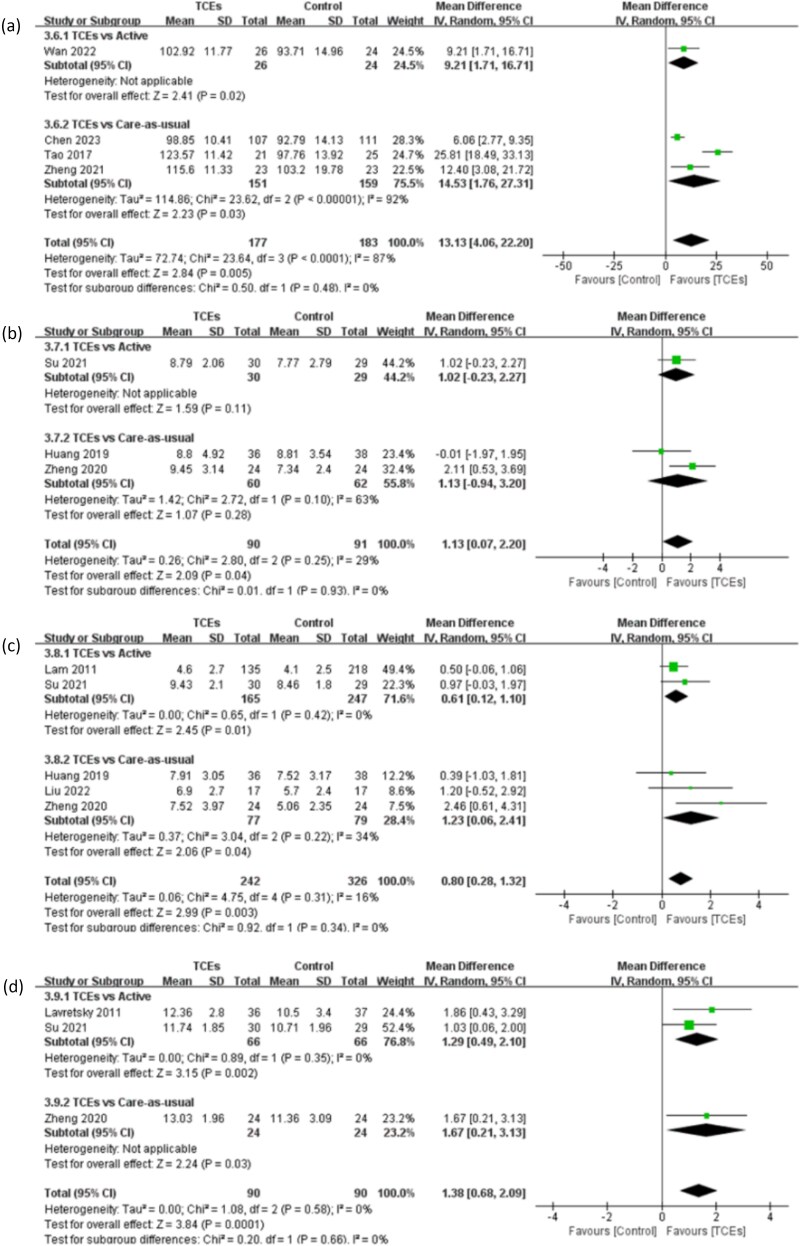
Forest plot of memory function. (a) Memory Quotient (MQ); (b) immediate recall of Auditory Verbal Learning Test (AVLT); (c) short-term delayed recognition of AVLT; (d) long-term delayed recognition of AVLT.

Four studies (*n* = 310) measured memory function using the MQ test [32]. Meta-analysis showed that TCE trainings had a significant positive effect on memory function [MD = 13.13; 95% CI: (4.06, 22.20); *P* = .005; Figure 5a], but there was high heterogeneity between studies (*P* < .0001; *I^2^* = 87%). Subgroup analyses revealed that the TCEs group performed better than both the active control group [MD = 9.21; 95% CI: (1.71, 16.71); *P* = .02] and the care-as-usual control group [MD = 14.53; 95% CI: (1.76, 27.31); *P* = .03].

Six studies reported the effects of TCEs on verbal memory measured using AVLT [33], including immediate recall in 181 participants, short-term delayed recognition in 833 participants, and long-term delayed recognition in 180 participants. TCE trainings improved verbal memory as shown by enhanced immediate recall [MD = 1.13; 95% CI: (0.07, 2.20); *P* = .04] with low heterogeneity (*P* = .25; *I^2^* = 29%) (Figure 5b), short-term delayed recognition [MD = 0.80; 95% CI: (0.28, 1.32); *P* = .003] with low heterogeneity (*P* = .31; *I^2^* = 16%) (Figure 5c) and long-term delayed recognition [MD = 1.38; 95% CI: (0.68, 2.09); *P* = .0001] with no heterogeneity (*P* = .58; *I^2^* = 0%) (Figure 5d). Subgroup analyses for the AVLT immediate recall subtest showed no significant difference between the TCEs group and the active control group [MD = 1.02; 95% CI: (−0.23, 2.27); *P* = .11].

All meta-analyses were conducted using random-effects models. The between-study variance (τ^2^) and 95% prediction intervals for each outcome are provided in Appendix S4.

### Sensitivity analyses

While the majority of pooled effect size remained stable, alteration of group effect sizes was observed in several domains. For the DS-F outcome, omitting Li et al., 2023 [29] reduced heterogeneity to *I^2^* = 37%, compared to original analysis (*I^2^* = 85%). Effect size of Digit Symbol Coding task (DSC) outcome, after omitting Chen et al., 2023 [19], reduced significantly [MD = 3.71; 95% CI: (−0.77, 8.20)], of which was no longer significant (*P* = .10). For the MQ outcome, omitting Tao et al., 2017 [34] eliminated heterogeneity (*I^2^* = 0%), compared to original analysis (*I^2^* = 85%). For the AVLT, omitting Zheng et al., 2020 [35] resulted in a non-significant effect for immediate recall (*P* = .18), and omitting Su et al., 2021 [36] led to moderate heterogeneity (*I^2^* = 63%) for immediate recall.

These findings suggest that the results for some cognitive outcomes, particularly DS-F, DSC, MQ and AVLT, may be influenced by the inclusion of specific studies. The observed changes in statistical difference and heterogeneity could be related to the number of included studies and the sample sizes. Details of the sensitivity analyses are presented in Appendix S5.

### Meta-regression analysis and publication bias

The meta- regression analysis, with 13 studies, revealed no significant effect of any of these variables on the MoCA scores except the covariate of type of TCE (Appendix S6), which suggested the type of TCE was the potential source of heterogeneity.

Publication bias was assessed for the MoCA outcome, which had a sufficient number of studies (*n* ≥ 10) for this analysis. Visual inspection of the funnel plot for MoCA indicated the presence of slight publication bias. However, Egger’s test did not detect statistically significant publication bias by (*P* = .231). Funnel plots of cognitive outcomes are presented in Appendix S7.

## Discussion

This meta-analysis of 28 RCTs, comprising 2297 participants, evaluated the effects of various types of TCE interventions on global cognition and several cognitive domains in older adults. Extending prior work [37], it includes a wider range of neuropsychological assessments and a more extensive literature search in adults ≥60 years. TCE training significantly improved global cognition, executive function, working memory, processing speed and memory, but not attention or visuospatial ability. Overall, preliminary to moderate evidence supports TCE as a promising strategy for cognitive enhancement in older adults, though methodological limitations and heterogeneity warrant confirmation in high-quality RCTs.

Cognitive decline is a key age-related change, commonly assessed using brief global cognition tests [38], such as the MoCA and the MMSE [39]. The MoCA is generally more sensitive to mild impairment, whereas the MMSE may show ceiling effects. Our pooled analysis revealed that TCEs significantly improved both MoCA and MMSE scores, suggesting potential benefit against global cognitive decline. The type of TCE was identified as a potential factor of heterogeneity in meta-regression, though subgroup analysis found no significant difference between subtypes—likely due to uneven study distribution rather than absence of effect. Additionally, greater MMSE improvement was observed with care-as-usual versus active control, a finding warranting further investigation.

Cognitive performance is commonly assessed using distinct domains in clinical neuropsychology [40]. Individual neuropsychological tests, categorised under different subdomains, can measure one or more discrete cognitive functions [41]. This meta-analysis examined six domains: executive function, working memory, processing speed, attention, memory function and visuospatial ability. TCEs training effectively improved most domains, except attention and visuospatial ability, which may be attributed to significant heterogeneity and the limited number of studies (four or fewer) included in the analyses. From a clinical perspective, subdomain changes are less clinically meaningful but may offer early insights and guide future research, and researchers should exercise caution when interpreting subdomain results.

Executive function, a multidimensional goal-directed system [42], is among the most complex cognitive processes, encompassing reasoning, problem solving, planning and other high-level activities [43]. This study demonstrated that TCE training positively impacts executive function. Notably, a significant study by Lin et al. [44] specifically evaluated the effects of exercise interventions on subdomains of executive function in older adults. This research provides valuable insights and paving the way for further systematic investigations into the role of TCEs in enhancing executive function and overall cognitive health.

This meta-analysis encompassing 28 RCTs with 2297 participants, provides an updated synthesis on TCE effects on cognitive function in adults aged 60 years and older. TCEs positively impact global cognition, executive function, working memory, processing speed and memory. Professional supervision is important to ensure correct technique and adherence. Future research should explore integration with other interventions (e.g. cognitive training, lifestyle programs). Compared to conventional exercise, TCEs offer practical advantages due to their low-impact nature and integration of movement, attention and breathing, which may support engagement for older adults. Simplified TCE protocols for older adults with cognitive impairment are also recommended. The pooled MD of 1.67 points on the MoCA approaches the Minimal Clinically Important Difference (MCID) (1.2–2.0 points) for older adults with mild cognitive impairment [45], suggesting potential clinical relevance in some subgroups, though MCID thresholds vary. Follow-up analyses were precluded by heterogeneity and limited studies, highlighting a gap for future research.

Limitations of this study needs to be mentioned. It focused exclusively on cognition-related outcomes; incorporating other frailty-related indicators (e.g. quality of life, fear of falling) could provide a more holistic perspective. We deviated from the PROSPERO protocol (≥50 vs. ≥60) to improve clinical homogeneity given the observed age heterogeneity across studies. The lack of reported adverse events and long-term follow-up data limits conclusions on safety and sustained effects of TCE training. High attrition rates and inadequate handling of missing data may compromise the validity of the estimated intervention effect. Heterogeneity due to variations in exercise interventions, assessment tools and health conditions of participants persisted despite subgroup analyses and meta-regressions were conducted. Future studies should prespecify subgroup analyses to avoid the risk of over-interpretation or bias. Finally, consistent with findings from a bibliometric analysis [46], most included studies were conducted by Chinese researchers, highlighting the need for more diverse geographic and ethnic representation.

## Conclusions

In conclusion, this study underscores the potential of TCEs as an effective, culturally rooted non-pharmacological intervention for improving cognitive function in older adults. The findings highlight promising benefits across various cognitive domains. To advance the field, future research should investigate long-term safety, effectiveness in diverse populations and integration with other interventions. Developing accessible, simplified TCE routines could further support healthy ageing worldwide.

## Supplementary Material

aa-25-1236-File002_afag168

## Key points

- Traditional Chinese exercises (TCEs) have a positive impact on cognitive function in adults aged 60 years and older.
- This meta-analysis showed that TCEs significantly improved global cognition, as well as several specific cognitive domains (executive function, working memory, processing speed and memory) in older adults, with or without other chronic diseases.
- Future research should evaluate long-term cognitive effects (>6 months), safety and adherence in frail older adults, functional outcomes and quality of life, and conduct large-scale RCTs in underrepresented groups (e.g. MCI, low education, diverse backgrounds).
