# Supplementary material for: Efficacy of traditional Chinese exercises on cognitive function in older adults: a systematic review and meta-analysis of randomised controlled trials
Source: Age Ageing. 2026 Jun 8;55(6):afag168. doi: 10.1093/ageing/afag168 (PMC13245186; doi:10.1093/ageing/afag168)
Supplement: aa-25-1236-File002_afag168 [file aa-25-1236-file002_afag168.docx]

**Efficacy of Traditional Chinese Exercises on cognitive function in older adults: A systematic review and meta-analysis of randomized controlled trials**

**Table of Contents**

Appendix S1, Search strategy

Table S1.

Appendix S2, Characteristics of included studies

Table S2.

Appendix S3, The risk of bias assessment for the included studies

Figure S1A and S1B.

Appendix S4, The between-study variance (τ²) and 95% prediction intervals

Table S3.

Appendix S5, Sensitivity analyses

Figure S2—— Figure S10.

Appendix S6, Summary of univariate moderator analyses in MoCA

Table S4.

Appendix S7,

Figure S11.

Appendix S8, Funnel plot

Figure S12—— Figure S15.

Appendix S9, GRADE summary

Table S5.

**Appendix S1, Search strategy**

Table S1

| Embase  (2024-5-2) | 1. 'cognitive defect'/exp 2. 'cognition disorders':ab,ti OR 'cognitive defects':ab,ti OR 'cognitive deficit':ab,ti OR 'cognitive disability':ab,ti OR 'cognitive disorders':ab,ti OR 'cognitive dysfunction':ab,ti OR 'cognitive impairment':ab,ti OR overinclusion:ab,ti OR 'response interference':ab,ti OR 'cognitive defect':ab,ti 3. #1 OR #2 4. 'aged'/exp 5. 'aged patient':ab,ti OR 'aged people':ab,ti OR 'aged person':ab,ti OR aged:ab,ti OR 'elderly patient':ab,ti OR 'elderly people':ab,ti OR 'elderly person':ab,ti OR 'elderly subject':ab,ti OR 'senior citizen':ab,ti OR senium:ab,ti 6. #4 OR #5 7. 'tai chi'/exp 8. 'tai chi chuan':ab,ti OR 'tai ji':ab,ti OR 'taiji quan':ab,ti OR taijiquan:ab,ti OR 'tai chi':ab,ti 9. 'qigong'/exp 10. 'chi kung':ab,ti OR chigung:ab,ti OR 'qi gong':ab,ti OR qigong:ab,ti 11. baduanjin:ab,ti OR liuzijue:ab,ti OR yijinjing:ab,ti OR wuqinxi:ab,ti OR taijisword:ab,ti OR 'taiji pushing hand':ab,ti OR dayangong:ab,ti OR daoyin:ab,ti OR 'traditional chinese exercise':ab,ti OR 'traditional chinese sports':ab,ti 12. #7 OR #8 OR #9 OR #10 OR #11 13. #3 AND #6 AND #12 |
| --- | --- |
| PubMed  (2024-5-2) | 1. "Cognitive Dysfunction"[Mesh] 2. (((((((((((((((((((((((((Cognitive Dysfunction[Title/Abstract]) OR (Cognitive Dysfunctions[Title/Abstract])) OR (Dysfunction, Cognitive[Title/Abstract])) OR (Dysfunctions, Cognitive[Title/Abstract])) OR (Cognitive Impairments[Title/Abstract])) OR (Cognitive Impairment[Title/Abstract])) OR (Impairment, Cognitive[Title/Abstract])) OR (Impairments, Cognitive[Title/Abstract])) OR (Cognitive Disorder[Title/Abstract])) OR (Cognitive Disorders[Title/Abstract])) OR (Disorder, Cognitive[Title/Abstract])) OR (Disorders, Cognitive[Title/Abstract])) OR (Mild Cognitive Impairment[Title/Abstract])) OR (Cognitive Impairment, Mild[Title/Abstract])) OR (Cognitive Impairments, Mild[Title/Abstract])) OR (Impairment, Mild Cognitive[Title/Abstract])) OR (Impairments, Mild Cognitive[Title/Abstract])) OR (Mild Cognitive Impairments[Title/Abstract])) OR (Cognitive Decline[Title/Abstract])) OR (Cognitive Declines[Title/Abstract])) OR (Decline, Cognitive[Title/Abstract])) OR (Declines, Cognitive[Title/Abstract])) OR (Mental Deterioration[Title/Abstract])) OR (Deterioration, Mental[Title/Abstract])) OR (Deteriorations, Mental[Title/Abstract])) OR (Mental Deteriorations[Title/Abstract]) 3. #1 OR #2 4. "Aged"[Mesh] 5. ((((((Aged[Title/Abstract]) OR (Elderly[Title/Abstract])) OR (elder[Title/Abstract])) OR (senior[Title/Abstract])) OR (older[Title/Abstract])) OR (retired[Title/Abstract])) OR (aging[Title/Abstract]) 6. #4 OR #5 7. "Tai Ji"[Mesh] 8. ((((((((((Tai-ji[Title/Abstract]) OR (Tai Ji[Title/Abstract])) OR (Tai Chi[Title/Abstract])) OR (Chi, Tai[Title/Abstract])) OR (Tai Ji Quan[Title/Abstract])) OR (Ji Quan, Tai[Title/Abstract])) OR (Quan, Tai Ji[Title/Abstract])) OR (Taiji[Title/Abstract])) OR (Taijiquan[Title/Abstract])) OR (T'ai Chi[Title/Abstract])) OR (Tai Chi Chuan[Title/Abstract]) 9. "Qigong"[Mesh] 10. ((Qigong[Title/Abstract]) OR (Qi Gong[Title/Abstract])) OR (Ch'i Kung[Title/Abstract]) 11. (((((((((Baduanjin[Title/Abstract]) OR (Liuzijue[Title/Abstract])) OR (Yijinjing[Title/Abstract])) OR (Wuqinxi[Title/Abstract])) OR (Taijisword[Title/Abstract])) OR (Taiji pushing hand[Title/Abstract])) OR (Dayangong[Title/Abstract])) OR (Daoyin[Title/Abstract])) OR (Traditional Chinese exercise[Title/Abstract])) OR (Traditional Chinese sports[Title/Abstract]) 12. #7 OR #8 OR #9 OR #10 OR #11 13. #3 AND #6 AND #12 |
| Cochrane  (2024-5-2) | 1. MeSH descriptor: [Cognitive Dysfunction] explode all trees 3988 2. (Cognitive Dysfunction):ti,ab,kw OR (Mild Cognitive Impairment):ti,ab,kw OR (Impairments, Mild Cognitive):ti,ab,kw OR (Impairment, Mild Cognitive):ti,ab,kw OR (Mild Cognitive Impairments):ti,ab,kw 13687 3. (Cognitive Impairments, Mild):ti,ab,kw OR (Cognitive Impairment, Mild):ti,ab,kw OR (Mental Deteriorations):ti,ab,kw OR (Deteriorations, Mental):ti,ab,kw OR (Cognitive Decline):ti,ab,kw 11730 4. (Decline, Cognitive):ti,ab,kw OR (Mental Deterioration):ti,ab,kw OR (Cognitive Declines):ti,ab,kw OR (Declines, Cognitive):ti,ab,kw OR (Deterioration, Mental):ti,ab,kw 8296 5. (Disorders, Cognitive):ti,ab,kw OR (Dysfunction, Cognitive):ti,ab,kw OR (Cognitive Disorder):ti,ab,kw OR (Impairments, Cognitive):ti,ab,kw OR (Cognitive Impairments):ti,ab,kw 44725 6. (Impairment, Cognitive):ti,ab,kw OR (Disorder, Cognitive):ti,ab,kw OR (Cognitive Dysfunctions):ti,ab,kw OR (Cognitive Disorders):ti,ab,kw OR (Cognitive Impairment):ti,ab,kw 48765 7. (Dysfunctions, Cognitive):ti,ab,kw 460 8. #1 OR #2 OR #3 OR #4 OR #5 OR #6 OR #7 56659 9. MeSH descriptor: [Aged] explode all trees 274545 10. (aged):ti,ab,kw OR (Elderly):ti,ab,kw OR (elder):ti,ab,kw OR (senior):ti,ab,kw OR (older):ti,ab,kw 705141 11. (retired):ti,ab,kw OR (aging):ti,ab,kw 19263 12. #9 OR #10 OR #11 710053 13. MeSH descriptor: [Tai Ji] explode all trees 582 14. (Tai Ji):ti,ab,kw OR (Taiji):ti,ab,kw OR (T'ai Chi):ti,ab,kw OR (Chi, Tai):ti,ab,kw OR (Tai Chi Chuan):ti,ab,kw 1923 15. (Ji Quan, Tai):ti,ab,kw OR (Taijiquan):ti,ab,kw OR (Tai Ji Quan):ti,ab,kw OR (Tai-ji):ti,ab,kw OR (Tai Chi):ti,ab,kw 1977 16. (Quan, Tai Ji):ti,ab,kw 36 17. MeSH descriptor: [Qigong] explode all trees 155 18. (Qigong):ti,ab,kw OR (Ch'i Kung):ti,ab,kw OR (Qi Gong):ti,ab,kw 810 19. (Baduanjin):ti,ab,kw OR (Liuzijue):ti,ab,kw OR (Yijinjing):ti,ab,kw OR (Wuqinxi):ti,ab,kw OR (Taijisword):ti,ab,kw 522 20. (Taiji pushing hand):ti,ab,kw OR (Dayangong):ti,ab,kw OR (Daoyin):ti,ab,kw OR (Traditional Chinese exercise):ti,ab,kw OR (Traditional Chinese sports):ti,ab,kw 725 21. #13 OR #14 OR #15 OR #16 OR #17 OR #18 OR #19 OR #20 3522 22. #8 AND #12 AND #21 256 |
| Web of Science  (2024-5-2) | 1. Cognitive Dysfunction (Topic) OR Cognitive Dysfunctions (Topic) OR Dysfunction, Cognitive (Topic) OR Cognitive Impairments (Topic) OR Cognitive Impairment (Topic) OR Impairment, Cognitive (Topic) OR Impairments, Cognitive (Topic) OR Cognitive Disorder (Topic) OR Cognitive Disorders (Topic) OR Disorder, Cognitive (Topic) OR Disorders, Cognitive (Topic) OR Mild Cognitive Impairment (Topic) OR Cognitive Impairment, Mild (Topic) OR Cognitive Impairments, Mild (Topic) OR Impairment, Mild Cognitive (Topic) OR Impairments, Mild Cognitive (Topic) OR Mild Cognitive Impairments (Topic) OR Cognitive Decline (Topic) OR Cognitive Declines (Topic) OR Decline, Cognitive (Topic) OR Declines, Cognitive (Topic) OR Mental Deterioration (Topic) OR Deterioration, Mental (Topic) OR Deteriorations, Mental (Topic) OR Mental Deteriorations (Topic) 2. Aged (Topic) OR Elderly (Topic) OR elder (Topic) OR senior (Topic) OR retired (Topic) OR aging (Topic) 3. Tai Ji (Topic) OR Tai-ji (Topic) OR Tai Chi (Topic) OR Chi, Tai (Topic) OR Tai Ji Quan (Topic) OR Ji Quan, Tai (Topic) OR Quan, Tai Ji (Topic) OR Taiji (Topic) OR Taijiquan (Topic) OR T'ai Chi (Topic) OR Tai Chi Chuan (Topic) OR Qigong (Topic) OR Qi Gong (Topic) OR Ch'i Kung (Topic) OR Baduanjin (Topic) OR Liuzijue (Topic) OR Yijinjing (Topic) OR Wuqinxi (Topic) OR Taijisword (Topic) OR Taiji pushing hand (Topic) OR Dayangong (Topic) OR Daoyin (Topic) OR Traditional Chinese exercise (Topic) OR Traditional Chinese sports (Topic) 4. #3 AND #2 AND #1 |
| Scopus  (2024-5-2) | 1. ( TITLE-ABS-KEY ( cognitive AND dysfunction ) OR TITLE-ABS-KEY ( cognitive AND dysfunctions ) OR TITLE-ABS-KEY ( dysfunction, AND cognitive ) OR TITLE-ABS-KEY ( dysfunctions, AND cognitive ) OR TITLE-ABS-KEY ( cognitive AND impairments ) OR TITLE-ABS-KEY ( cognitive AND impairment ) OR TITLE-ABS-KEY ( impairment, AND cognitive ) OR TITLE-ABS-KEY ( impairments, AND cognitive ) OR TITLE-ABS-KEY ( cognitive AND disorder ) OR TITLE-ABS-KEY ( cognitive AND disorders ) OR TITLE-ABS-KEY ( disorder, AND cognitive ) OR TITLE-ABS-KEY ( disorders, AND cognitive ) OR TITLE-ABS-KEY ( mild AND cognitive AND impairment ) OR TITLE-ABS-KEY ( cognitive AND impairment, AND mild ) OR TITLE-ABS-KEY ( cognitive AND impairments, AND mild ) OR TITLE-ABS-KEY ( impairment, AND mild AND cognitive ) OR TITLE-ABS-KEY ( impairments, AND mild AND cognitive ) OR TITLE-ABS-KEY ( mild AND cognitive AND impairments ) OR TITLE-ABS-KEY ( cognitive AND decline ) OR TITLE-ABS-KEY ( cognitive AND declines ) OR TITLE-ABS-KEY ( decline, AND cognitive ) OR TITLE-ABS-KEY ( declines, AND cognitive ) OR TITLE-ABS-KEY ( mental AND deterioration ) OR TITLE-ABS-KEY ( deterioration, AND mental ) OR TITLE-ABS-KEY ( deteriorations, AND mental ) ) 2. ( TITLE-ABS-KEY ( frail AND elderly ) OR TITLE-ABS-KEY ( elderly, AND frail ) OR TITLE-ABS-KEY ( frail AND elders ) OR TITLE-ABS-KEY ( elder, AND frail ) OR TITLE-ABS-KEY ( elders, AND frail ) OR TITLE-ABS-KEY ( frail AND elder ) OR TITLE-ABS-KEY ( functionally-impaired AND elderly ) OR TITLE-ABS-KEY ( elderly, AND functionally-impaired ) OR TITLE-ABS-KEY ( functionally AND impaired AND elderly ) OR TITLE-ABS-KEY ( frail AND older AND adults ) OR TITLE-ABS-KEY ( adult, AND frail AND older ) OR TITLE-ABS-KEY ( adults, AND frail AND older ) OR TITLE-ABS-KEY ( frail AND older AND adult ) OR TITLE-ABS-KEY ( older AND adult, AND frail ) OR TITLE-ABS-KEY ( older AND adults, AND frail ) OR TITLE-ABS-KEY ( aged ) OR TITLE-ABS-KEY ( elder ) OR TITLE-ABS-KEY ( senior ) OR TITLE-ABS-KEY ( older ) OR TITLE-ABS-KEY ( retired ) OR TITLE-ABS-KEY ( aging ) ) 3. 1# AND 2# 4. ( TITLE-ABS-KEY ( tai AND ji ) OR TITLE-ABS-KEY ( tai-ji ) OR TITLE-ABS-KEY ( tai AND chi ) OR TITLE-ABS-KEY ( chi, AND tai ) OR TITLE-ABS-KEY ( tai AND ji AND quan ) OR TITLE-ABS-KEY ( ji AND quan, AND tai ) OR TITLE-ABS-KEY ( quan, AND tai AND ji ) OR TITLE-ABS-KEY ( taiji ) OR TITLE-ABS-KEY ( taijiquan ) OR TITLE-ABS-KEY ( t'ai AND chi ) OR TITLE-ABS-KEY ( tai AND chi AND chuan ) OR TITLE-ABS-KEY ( qigong ) OR TITLE-ABS-KEY ( qi AND gong ) OR TITLE-ABS-KEY ( ch'i AND kung ) OR TITLE-ABS-KEY ( baduanjin ) OR TITLE-ABS-KEY ( liuzijue ) OR TITLE-ABS-KEY ( yijinjing ) OR TITLE-ABS-KEY ( wuqinxi ) OR TITLE-ABS-KEY ( taijisword ) OR TITLE-ABS-KEY ( taiji AND pushing AND hand ) OR TITLE-ABS-KEY ( dayangong ) OR TITLE-ABS-KEY ( daoyin ) OR TITLE-ABS-KEY ( traditional AND chinese AND exercise ) OR TITLE-ABS-KEY ( traditional AND chinese AND sports ) ) 5. ( TITLE-ABS-KEY ( randomized AND controlled AND trials AND as AND topic ) OR TITLE-ABS-KEY ( clinical AND trials, AND randomized ) OR TITLE-ABS-KEY ( trials, AND randomized AND clinical ) OR TITLE-ABS-KEY ( controlled AND clinical AND trials, AND randomized ) OR TITLE-ABS-KEY ( randomized AND controlled AND trial ) OR TITLE-ABS-KEY ( controlled AND clinical AND trial ) OR TITLE-ABS-KEY ( rct ) ) 6. #3 AND #4 AND #5 |
| PsycINFO  (2024-5-2) | 1. (Cognitive Dysfunction or Cognitive Dysfunctions or Dysfunction, Cognitive or Dysfunctions, Cognitive or Cognitive Impairments or Cognitive Impairment or Impairment, Cognitive or Impairments, Cognitive or Cognitive Disorder or Cognitive Disorders or Disorder, Cognitive or Disorders, Cognitive or Mild Cognitive Impairment or Cognitive Impairment, Mild or Cognitive Impairments, Mild or Impairment, Mild Cognitive or Impairments, Mild Cognitive or Mild Cognitive Impairments or Cognitive Decline or Cognitive Declines or Decline, Cognitive or Declines, Cognitive or Mental Deterioration or Deterioration, Mental or Deteriorations, Mental or Mental Deteriorations).af. 219023 2. (Aged or Elderly or elder or senior or older or retired or aging).af. 1342805 3. (Tai Ji or Tai-ji or Tai Chi or Chi, Tai or Tai Ji Quan or Ji Quan, Tai or Quan, Tai Ji or Taiji or Taijiquan or T'ai Chi or Tai Chi Chuan).af. 4442 4. (Qigong or Qi Gong or Ch'i Kung or Baduanjin or Liuzijue or Yijinjing or Wuqinxi or Taijisword or Taiji pushing hand or Dayangong or Daoyin or Traditional Chinese exercise or Traditional Chinese sports).af. 2311 5. 3 or 4 5758 6. 1 and 2 and 5 1167 |

**Appendix S2, The characteristics of the included studies**

Table S2

| **Study** | **Country/**  **Location** | **Health condition** | **Total N (intervention group, control group)** | **Mean age** | **Sex (% female)** | **Type of TCEs** | **mode of instruction** | **Characteristic of control** | **Duration** | **Sessions/week** | **Session length** | **Outcome measurements** | **no. of arms** | **Numbers Lost to Follow-up** | **Handling of Missing Data** |
| --- | --- | --- | --- | --- | --- | --- | --- | --- | --- | --- | --- | --- | --- | --- | --- |
| **Angus P. Yu et al., 2022** | Hong Kong, China | MCI | 22(10,12) | 67.4 | 68.18 | Tai Chi | Coaches | Active (fitness training) | 24 weeks | 3 | 60 min | MoCA, 30-min Delay Recall, DS- F, DS-B, TMT-A/B, Victoria Stroop Test, N-back Task | 3 | 3, 8.11% | CCA, GEE |
| **Chan et al., 2016** | Hong Kong, China | MCI | 52(27,25) | 80.3 | 84.62 | tai chi | tai chi master | Care-as-usual | 2 months | 2 | 60 min | MMSE, MIC | 2 | 21, 40.38% | ITT, GEE |
| **Chen et al., 2023** | Fuzhou, China | T2D and MCI | 218(107,111) | 67.55 | 50.91 | Tai Chi | Coaches | Care-as-usual | 24 weeks | 3 | 60 min | MoCA, WMS-MQ, DSC, TMT-B, BNT, ROCF | 3 | 46, 14.02% | MI |
| **Cheng et al., 2014** | Hong Kong, China | dementia | 110(75,35) | 81.55 | 64.5 | seated Tai Chi | Unknown | Active  (simple handicrafts) | 12 weeks | 3 | 60 min | CDR-SB | 3 | 0 | ITT, MMER, FIML |
| **Gerritsen et al., 2021** | Leiden, The Netherlands | Normal | 43(20,23) | 63.53 | 46.51 | Tai Chi | online video | Care-as-usual | 10 weeks | 2 | 45 min | DSST, TMT, SCWT, MMSE, MoCA | 2 | 0 | CCA, B-rmANOVA |
| **Huang et al., 2019** | Beijing, China | mild dementia | 74(36,38) | 82 | 67.5 | Tai Chi | Coaches | Care-as-usual | 10 months | 3 | 20 min | MMSE, MoCA, AVLT, TMT | 2 | 6, 7.5% | CCA, GEE |
| **Hwang et al., 2023** | Taiwan, China | MCI | 84(35,49) | ≥65 | Unknown | Yang-style Tai Chi | professional instructors | Active (physical activities) | 6 months | 1 | 50 min | MDRS | 2 | 30, 15.87% | ITT, LMER |
| **Jin et al., 2020** | Shanghai, China | community-dwelling older adults | 51(25, 26) | 66.1 | 84.3 | Qigong | Video | Active  (core exercise) | 12 weeks | 2 | 60 min | MoCA and RBANS | 2 | 15, 22.73% | ITT, GEE, rmANOVA |
| **Lam et al., 2011** | Hong Kong, China | MCI | 353(135,218) | 77.75 | 76.35 | Tai Chi | Coaches | Active (stretching and toning exercise) | 24 weeks | 3 | 30 min | CDR, MIC, ADAS-cog, Digit Span, Visual Span, TMT-A/B, Verbal Fluency, MMSE, delayed recall | 2 | 60, 15.42% | ITT |
| **Lavretsky et al., 2011** | Los Angeles; USA | geriatric depression | 73(36,37) | 70.6 | 61.6 | Tai Chi | Unclear | Active (health education) | 10 weeks | 1 | 120 min | MMSE, CVLT long delayed recall, Trails A errors | 2 | 7, 6.25% | ITT, ML |
| **Li et al., 2023** | Springfield, USA | MCI | 213(107,106) | 76 | 66.7 | Tai Chi | Online Zoom | Active (stretching exercise) | 24 weeks | 2 | 60 min | MoCA, Dual-task costs, CDR-SB, TMT-B, Verbal Fluency, DS-F, DS-B | 3 | 14, 4.40% | ITT, MI |
| **Lin et al., 2023** | Fuzhou, China | cognitive frailty | 102(51,51) | 66.52 | 61.8 | Baduanjin | Coaches | Active (health education) | 24 weeks | 3 | 60 min | MoCA | 2 | 6, 5.88% | ITT, MI |
| **Liu et al., 2019** | Fuzhou, China | Normal | 33(23,10) | 61.29 | 67.2 | Tai Chi and Baduanjin | professional instructors | Care-as-usual | 12 weeks | 5 | 60 min | MQ, WMS-CR | 3 | 0 | CCA |
| **Liu et al., 2022** | Taiwan, China | MCI | 34(17,17) | 73.5 | 70 | Tai Chi | Coaches | Care-as-usual | 12 weeks | 3 | 50 min | MoCA, TMT(B-A), CVLT, SCWT, N-back Tasks | 3 | 0 | CCA |
| **Lipsitz et al., 2019** | Boston, USA | multiple chronic conditions without dementia | 180(93,87) | 75.3 | 66.7 | Tai Chi | Coaches | Active (health education) | 52 weeks | 3 | 20 min | TMT(B-A) | 2 | 5, 2.78% | ITT, SBIM |
| **Luo et al., 2022** | Anqing, China | MCI | 48(24,24) | 65.9 | 79.17 | Wuqinxi | Coaches | Care-as-usual | 40 weeks | 1 | 60 min | N-back Tasks | 2 | 0 | CCA |
| **Moon et al., 2020** | Kansas City, USA | Parkinson`s disease | 17(8,9) | 66.15 | 41.18 | Six Healing Sounds | Coaches | Active (physical activities) | 12 weeks | 7 | 40 min | FAB, CDT, TMT-A/B | 2 | 4, 12.5% | CCA |
| **Mortimer 2012 et al.,** | Shanghai, China | Normal | 53(29,24) | 67.8 | 66.7 | Tai Chi | certified Tai Chi master | Care-as-usual | 40 weeks | 2 | 50 min | MDRS, TMT, AVLT |  | 13, 10.83% | ITT, RMM |
| **Oh et al., 2012** | Sydney, Australia | cancer patients | 54(23,31) | 62.69 | 50 | Qigong | experienced instructor | Care-as-usual | 10 weeks | 2 | 90 min | EORTC QLQ-C30 CF, FACT-Cog |  | 23, 28.39% | CCA |
| **Qi et al., 2021** | Hong Kong, China | non-dementia (MoCA＞22) | 48(22,26) | 64.23 | 68.8 | Wu Xing Ping Heng Gong | Coaches | Active (stretching exercise) | 12 weeks | 2 (first 6 weeks),1 (last 6 weeks) | 120 min | SDMT, CPT 3, DS-B | 2 | 0 | CCA, LMM |
| **Qi et al., 2024** | Dalian, China | Normal | 20 | 62.8 | 55 | Tai chi Quan (28 simplified chen style moves) | Professional coaches | Active, Brisk walking | 1 weeks | 1 | 6 min | WCST | 3 | 0 | CCA |
| **Redwine et al., 2020** | San Diego, USA | heart failure | 47(24,23) | 65 | 21 | Tai Chi | Coaches | Care-as-usual | 16 weeks | 2 | 60 min | MoCA | 3 | 1, 1.43% | ITT, ME |
| **Siu et al., 2018** | Hong Kong, China | MCI | 160(80,80) | ≥60 | 73.8 | Tai Chi | Coaches | Care-as-usual | 16 weeks | 2 | 60 min | MMSE | 2 | 3, 1.88% | CCA, GEE |
| **Su et al., 2021** | Harbin, China | non-dementia | 59(30,29) | 64.9 | 55.38 | Baduanjin | Coaches | Active (gymnastics practice) | 12 weeks | 5 | 60 min | AVLT, TMT-A/B, | 2 | 7, 8.75% | CCA |
| **Sungkarat et al., 2018** | Chiang Mai, Thailand | MCI | 66(33,33) | 67.9 | 86.4 | Tai Chi | Video | Active (health education) | 6 months | 3 | 50 min | LM-delayed recall, BDT, DS-F, DS-B, TMT(B-A) | 2 | 0 | ITT, MI |
| **Tao et al., 2017** | Fuzhou, China | non-dementia (MMSE≥24) | 46(21,25) | 61.07 | 70 | Tai Chi | Coaches | Care-as-usual | 12 weeks | 5 | 60 min | WMS-MQ | 3 | 0 | CCA |
| **Tao et al., 2017** | Fuzhou, China | normal, MMSE≥24 | 34(24,10) | 61.45 | 67.21 | Tai Chi and Baduanjin | professional instructors | Care-as-usual | 12 weeks | 5 | 60 min | WMS-CR, MQ, VRS | 3 | 0 | CCA |
| **Tao et al., 2019** | Fuzhou, China | MCI | 40(20,20) | 65.49 | 66.1 | Baduanjin | Coaches | Active (brisk walking) | 24 weeks | 3 | 60 min | MoCA | 3 | 2, 2.89% | CCA |
| **Tsai et al., 2013** | Little Rock, USA | cognitive impairment and knee OA | 55(28,27) | 78.91 | 72.7 | Tai Chi | Coaches | Active (health education) | 20 weeks | 3 | 20 min | MMSE | 2 | 0 | ITT, GIM |
| **Walsh et al., 2015** | Boston, USA | non-dementia (MMSE＞24) | 60(31,29) | 64.2 | 66.7 | Tai Chi | Coaches | Care-as-usual | 6 months | 4 | 30 min | DS-F, DS-B, COWAT, TMT-A/B | 2 | 3, 3.45% | ITT, RS |
| **Wan et al., 2022** | Fuzhou, China | MCI | 50(26,24) | 66.06 | 58 | Baduanjin | Coaches | Active (health education) | 24 weeks | 3 | 60 min | MoCA, WMS-MQ | 2 | 9, 8.82% | ITT, MI |
| **Wang et al., 2023** | Beijing, China | non-dementia (MMSE＞24) | 47(23,24) | 66.34 | Unclear | Tai Chi | Coaches | Care-as-usual | 12 weeks | 3 | 60 min | N-back Tasks | 2 | 0 | CCA |
| **Xia et al., 2019** | Fuzhou, China | MCI | 46(23,23) | 65.51 | 66.7 | Baduanjin | Coaches | Care-as-usual | 24 weeks | 3 | 60 min | CWMS, TAP, DSC | 3 | 0 | CCA |
| **Xia 2023 et al.,** | Fuzhou, China | MCI | 77(36,41) | 66.12 | 55.6 | Baduanjin | Coaches | Active, brisk walking | 24 weeks | 3 | 60 min | MoCA, TMT, DSC, AVLT | 3 | 8, 5.93% | ITT, MI |
| **Ye Yu et al., 2022** | Changsha, China | non-dementia (MoCA＞26) | 43(21,22) | 65.88 | 55.81 | Tai Chi | Coaches | Active (brisk walking) | 10 weeks | 3 | 60 min | MoCA | 2 | 0 | CCA |
| **Zhang et al., 2022** | Daqing, China | cognitive frailty | 61(31,30) | 71.3 | 56.04 | Tai Chi | Coaches | Active (meditation practices) | 6 months | 2 | 60 min | MMSE | 3 | 2, 2.15% | ITT, rmANOVA |
| **Zheng et al., 2020** | Fuzhou, China | post-stroke cognitive impairment | 48(24,24) | 62.19 | 14.6 | Baduanjin | Coaches | Care-as-usual | 24 weeks | 3 | 40 min | MoCA, TMT-A/B, AVLT, TAP, DSC, CDT | 2 | 13, 27.08% | ITT, MI, LMM |
| **Zheng et al., 2021** | Fuzhou, China | MCI | 46(23,23) | 65.51 | 66.7 | Baduanjin | Coaches | Care-as-usual | 24 weeks | 3 | 60 min | MoCA, WMS-MQ, memory function subscores | 3 | 0 | CCA |
| **Zhu et al., 2020** | Hangzhou, China | Parkinson`s disease | 41(19,22) | 68.15 | 39.02 | Tai Chi | Coaches | Active (routine exercise) | 12 weeks | 3 | 50 min | MoCA | 2 | 0 | ITT, SAI |

ADAS-cog: Alzheimer's Disease Assessment Scale-Cognitive section; AVLT: Auditory Verbal Learning Test; BDT: Block Design Test; B-rmANOVA: Bayesian repeated-measures ANOVA; CCA: Complete-case analysis; CDR-SB: CDR sum-of-box; CDT: Clock Drawing Task; COWAT: the Controlled Oral Word Association Test; CPT 3: The Conners Continuous Performance Test 3rd edition; CWMS: Color-Word Matching Stroop task; CVLT: California Verbal Learning Test; DSC: Digit Symbol Coding task; DR-SB: Clinical Dementia Rating-Sum of Boxes; DS-B: Digit Span-Backwards; DS-F: Digit Span-Forwards; DSST: Digit Symbol Substitution Test; EORTC QLQ-C30 CF: European Organization for Research and Treatment of Cancer; FAB: Frontal Assessment Battery; FACT-Cog: Functional Assessment of Cancer Therapy—Cognitive Function; FIML: Full Information Maximum Likelihood; GEE: Generalized Estimating Equations; GIM: General linear mixed models; ITT: Intention-to-Treat analysis; LM: Logical Memory; LMM: linear mixed model; LMER: Linear mixed-effect regression model; MDRS: Mattis Dementia Rating Scale; MCI: mild cognitive impairment; MI:Multiple imputation; MIC: Memory Inventory for the Chinese; ML: Mixed linear models; MMER: Multilevel mixed-effects regression model; MMSE: Mini-Mental State Examination; MCI: Mild Cognitive Impairment; MoCA: Montreal Cognitive Assessment; ME: Mixed-effects model; OA: Osteoarthritic; RBANS: Repeatable Battery for the Assessment of Neuropsychological Status; RMM: Repeated-measures mixed models; rmANOVA: Repeated Measures ANOVA; ROCF: Rey-Osterrieth Complex Figure Test; RS: Random-slopes model; SAI: Simple assignment imputation; SBIM:Shared-baseline linear mixed; SCWT: Stroop Color and Word Test; SDMT: Symbol Digit Modality Test; T2D: Type 2 Diabetes; TAP: Test of Attention Performance; TMT: Trail Making Test; VRS: Visual Reproduction Subscores; WMS-CR: Wechsler Memory Scale-Chinese Revised; WMS-MQ: Wechsler Memory Scale- Memory Quotient

**Appendix S3, The risk of bias assessment for the included studies**


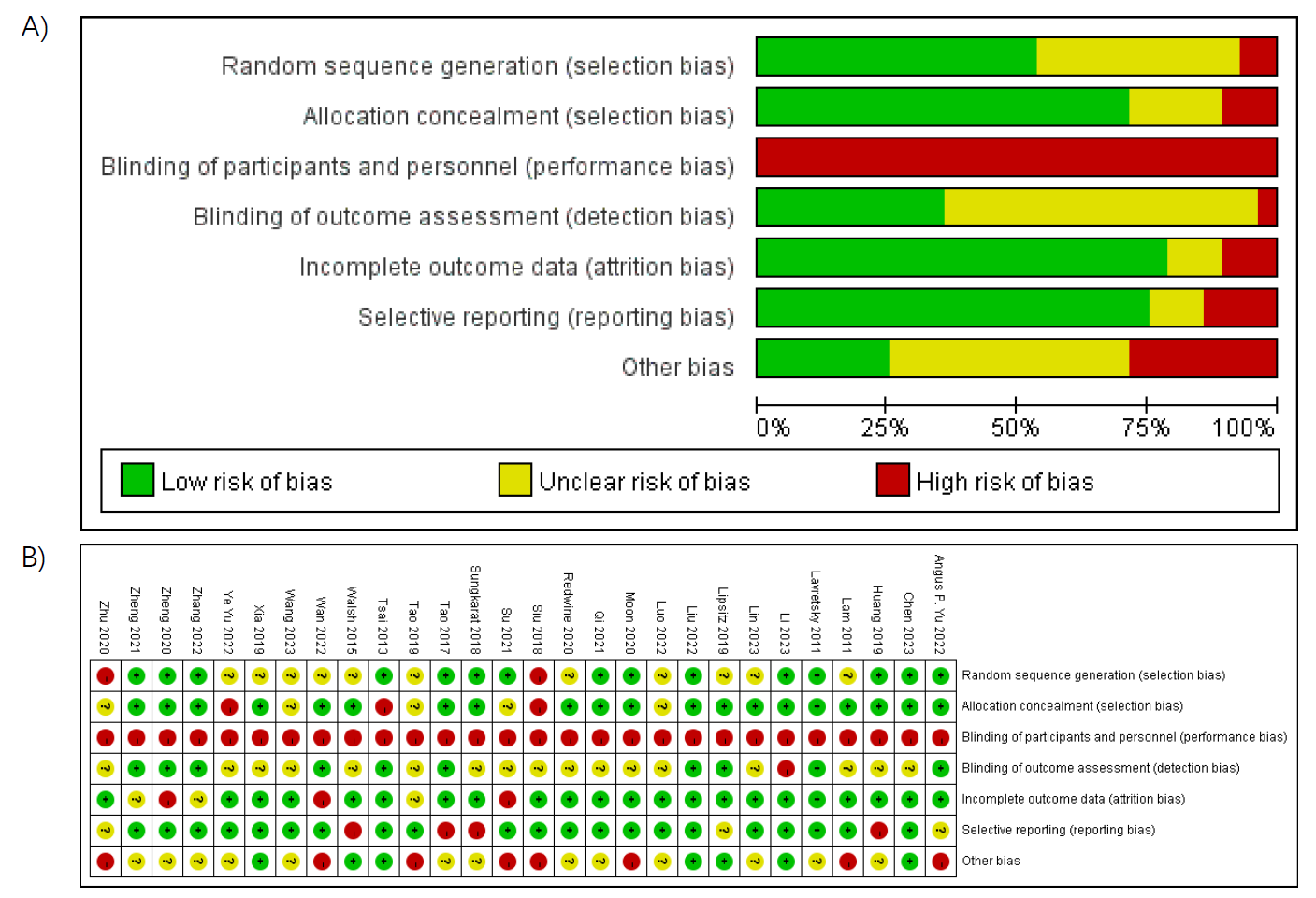


**Figure S1.** A) Risk of bias graph; B) Risk of bias summary.

**Appendix S4, The between-study variance (τ²) and 95% prediction intervals for each outcome**

Table S3

| Outcome | Number of Studies | τ² | 95% Prediction Interval |
| --- | --- | --- | --- |
| Montreal Cognitive Assessment (MoCA) | 13 | 0.29 | 0.252, 3.123 |
| Mini-Mental State Examination (MMSE) | 7 | 0.63 | -1.288, 2.804 |
| Trail Making Test (TMT) (B-A) | 9 | 47.79 | -30.038, 12.814 |
| Category Fluency for Animals | 3 | 0.00 | 0.465, 5.376 |
| Digit Span-Backwards (DS-B) | 5 | 0.10 | -0.612, 1.580 |
| Digit Symbol Coding (DSC) | 3 | 0.00 | -0.978, 9.301 |
| Digit Span-Forwards (DS-F) | 4 | 0.54 | -2.446, 3.393 |
| Clock Drawing Task (CDT) | 2 | 0.03 | -3.468, 3.408 |
| Memory Quotient (MQ) | 4 | 72.74 | -16.433, 42.656 |
| immediate recall of Auditory Verbal Learning Test (AVLT) | 3 | 0.26 | -1.637, 3.915 |
| short-term delayed recognition of AVLT | 5 | 0.06 | -0.032, 1.557 |
| long-term delayed recognition of AVLT | 3 | 0.00 | -0.166, 2.928 |

**Appendix S5, Sensitivity analyses**


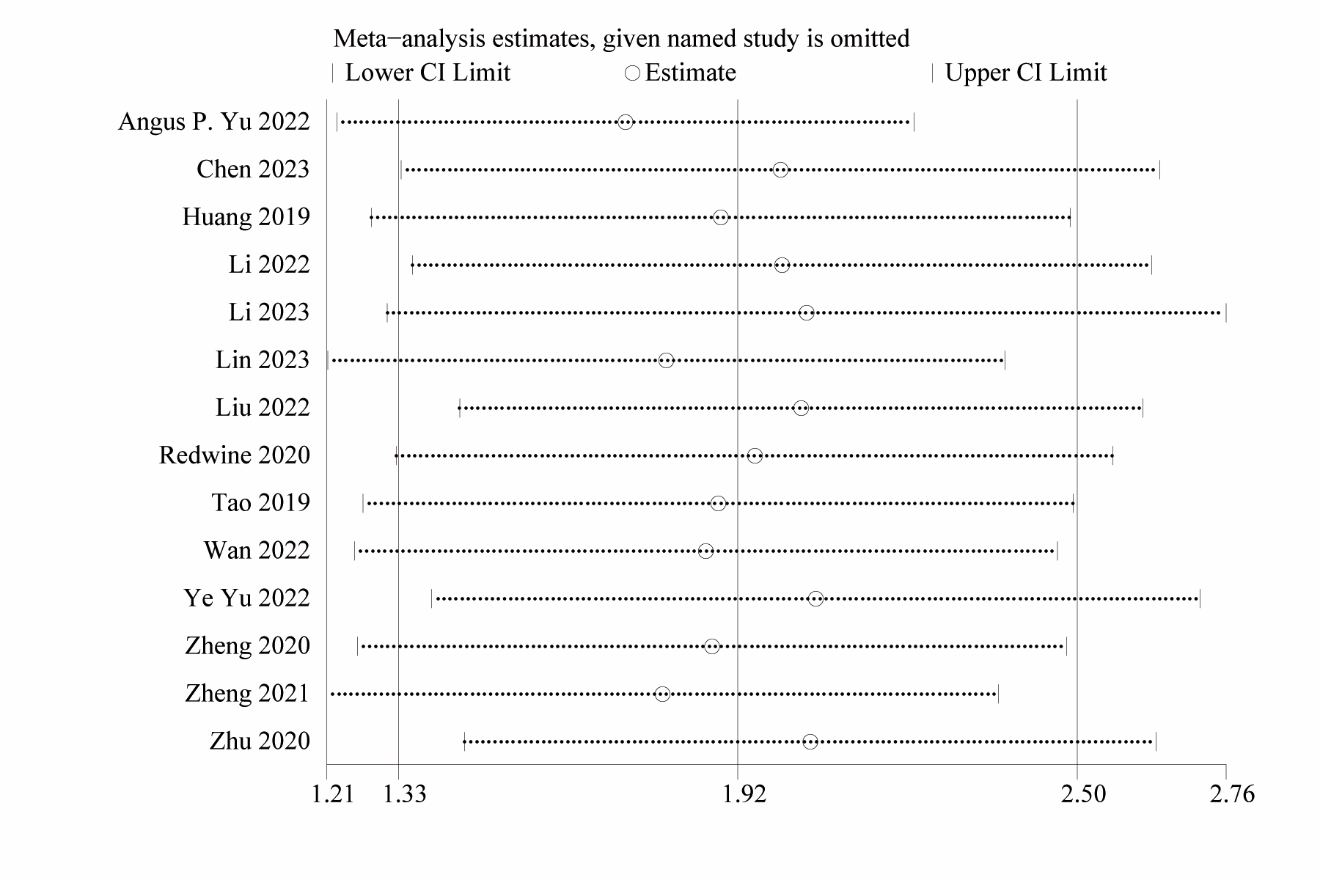
**Figure S2.** Sensitivity analyses for MoCA.


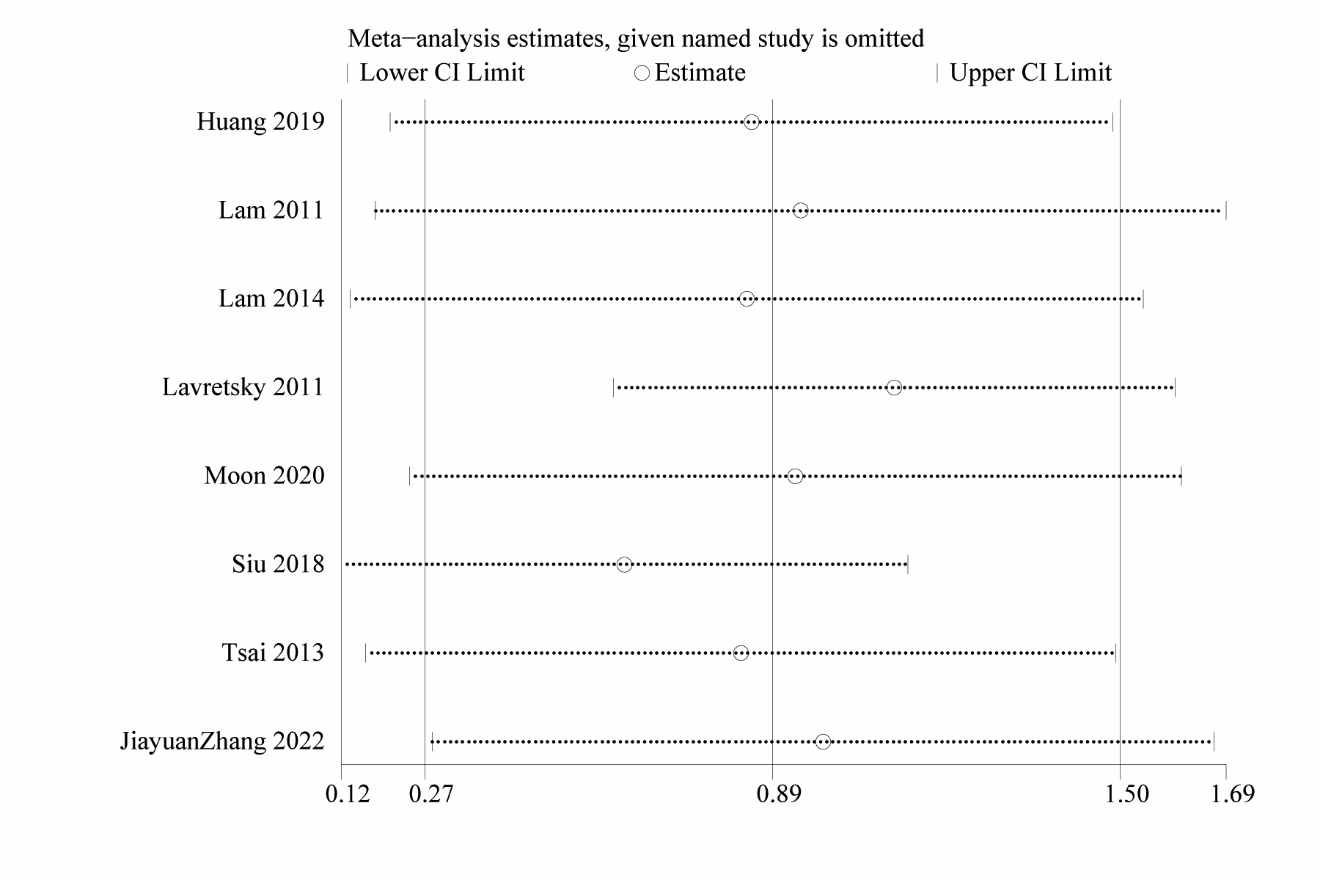


**Figure S3.** Sensitivity analyses for MMSE.


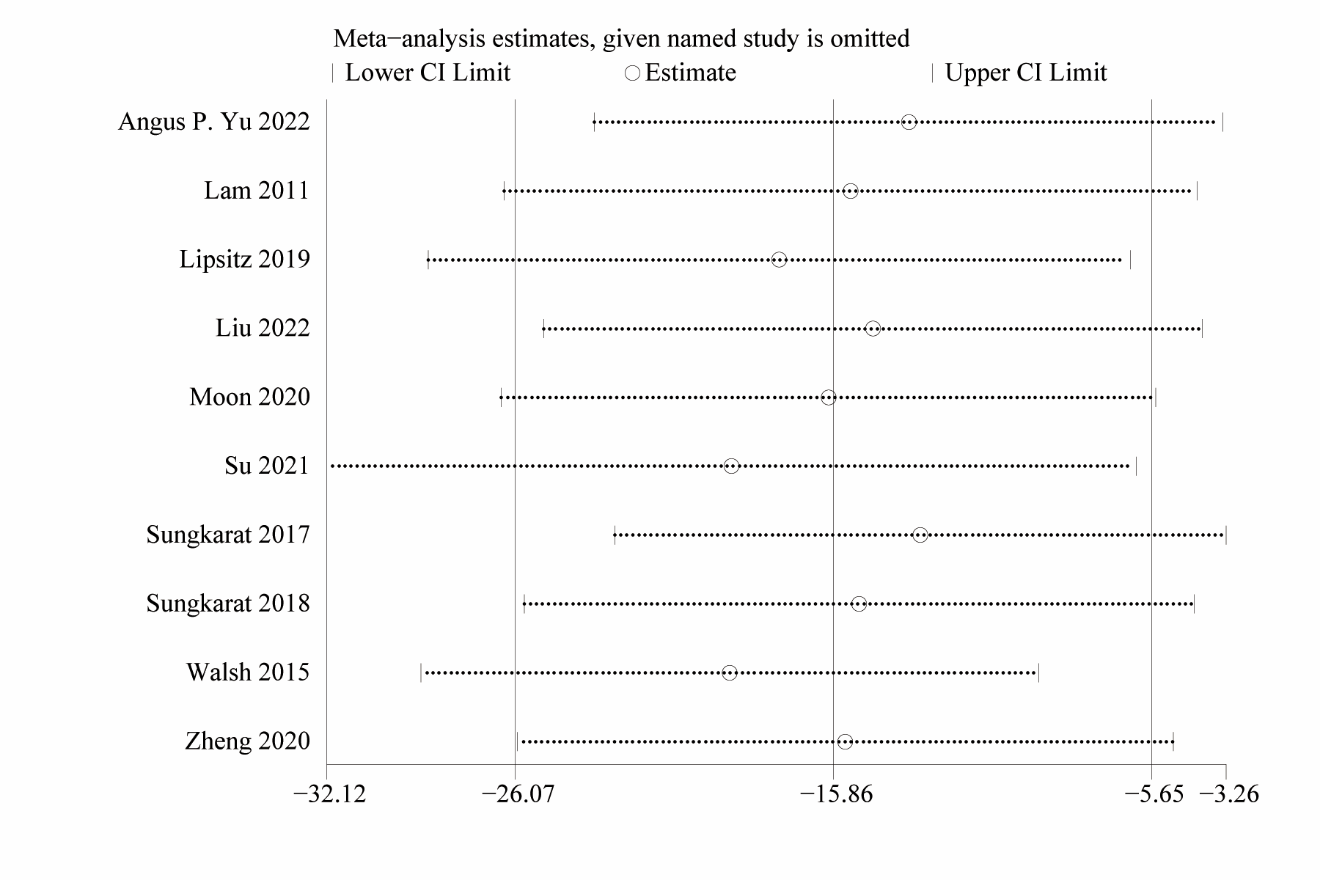


**Figure S4.** Sensitivity analyses for TMT (B-A).


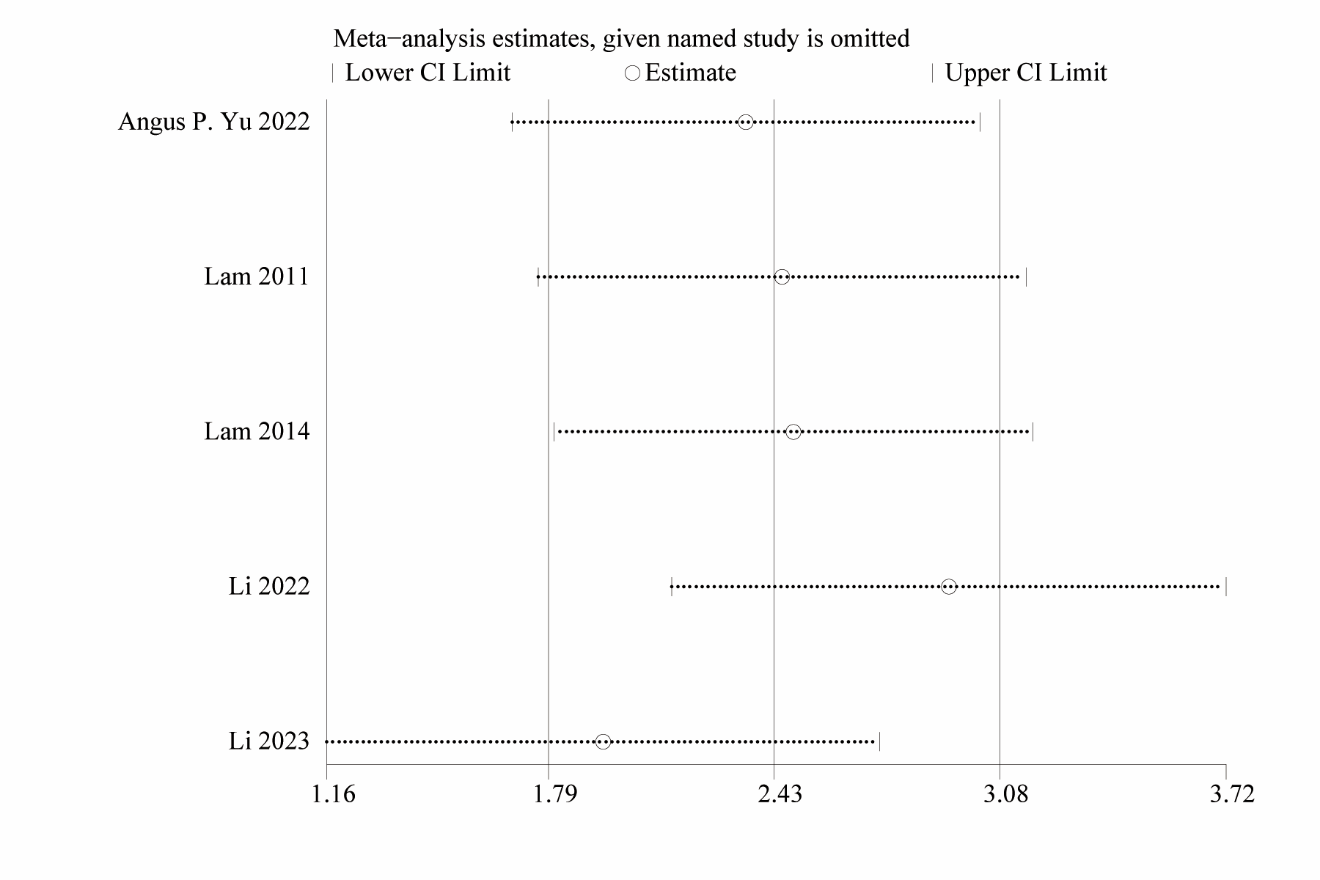


**Figure S5.** Sensitivity analyses for Verbal Fluency.


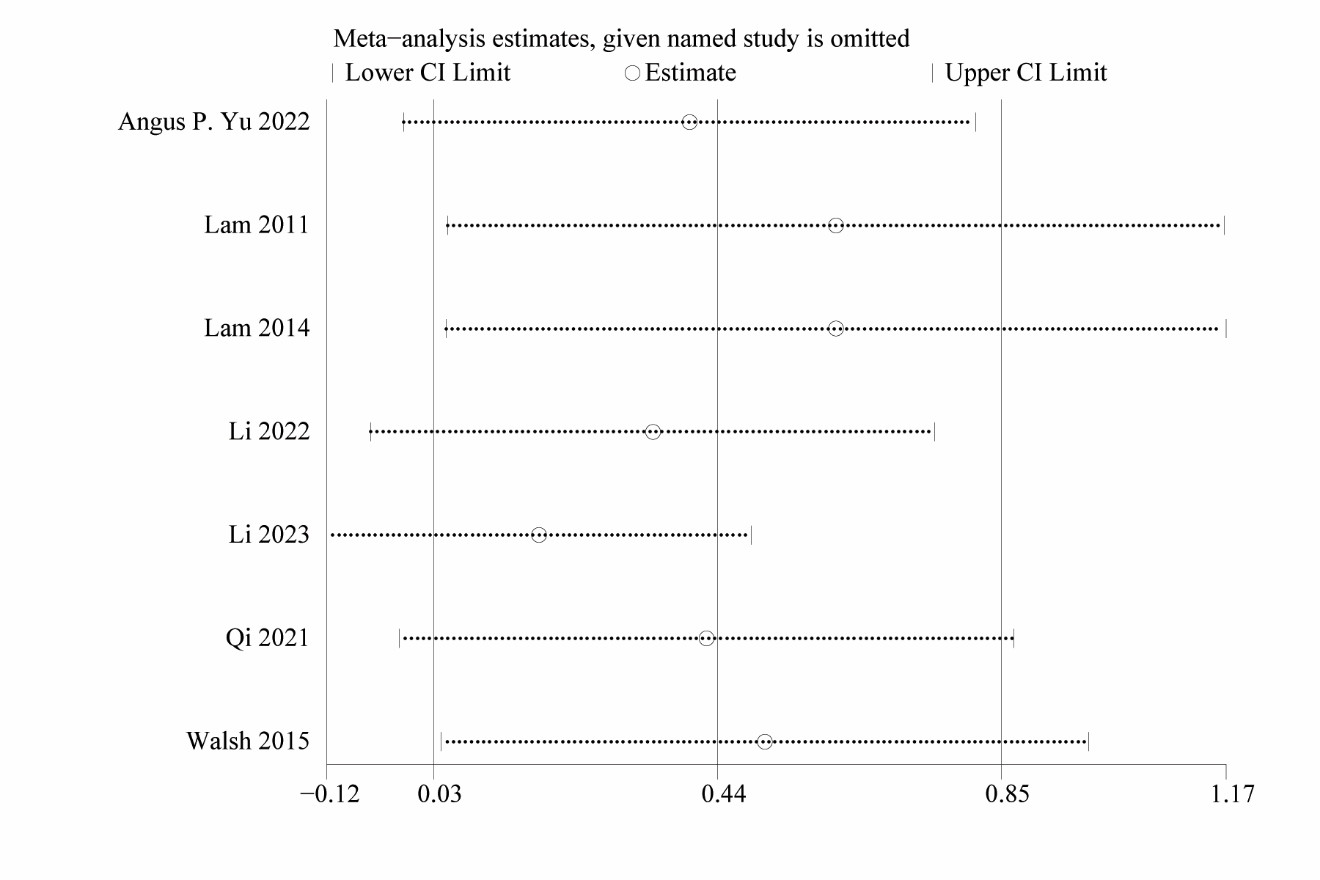


**Figure S6.** Sensitivity analyses for DS-B.


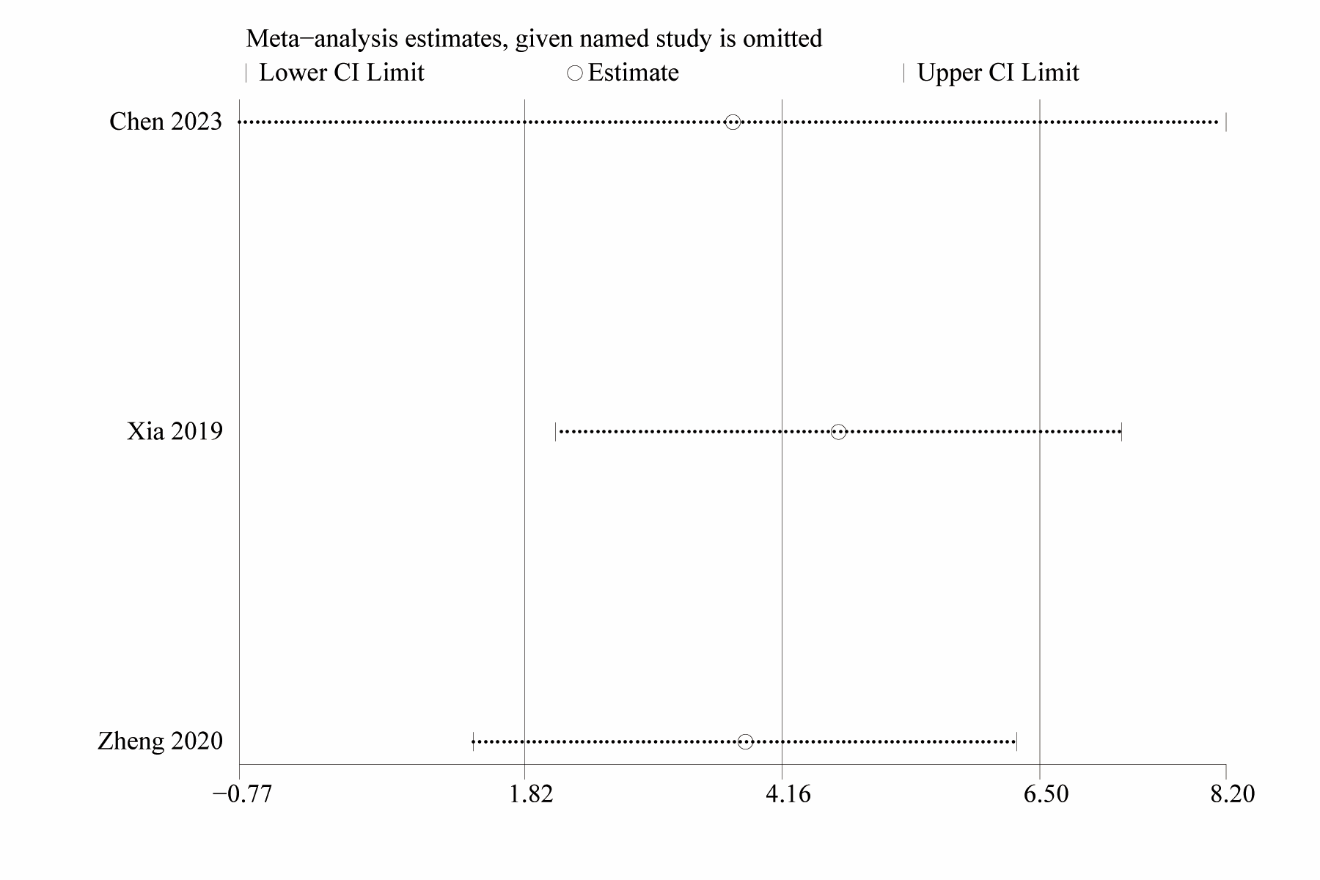


**Figure S7.** Sensitivity analyses for DSC.


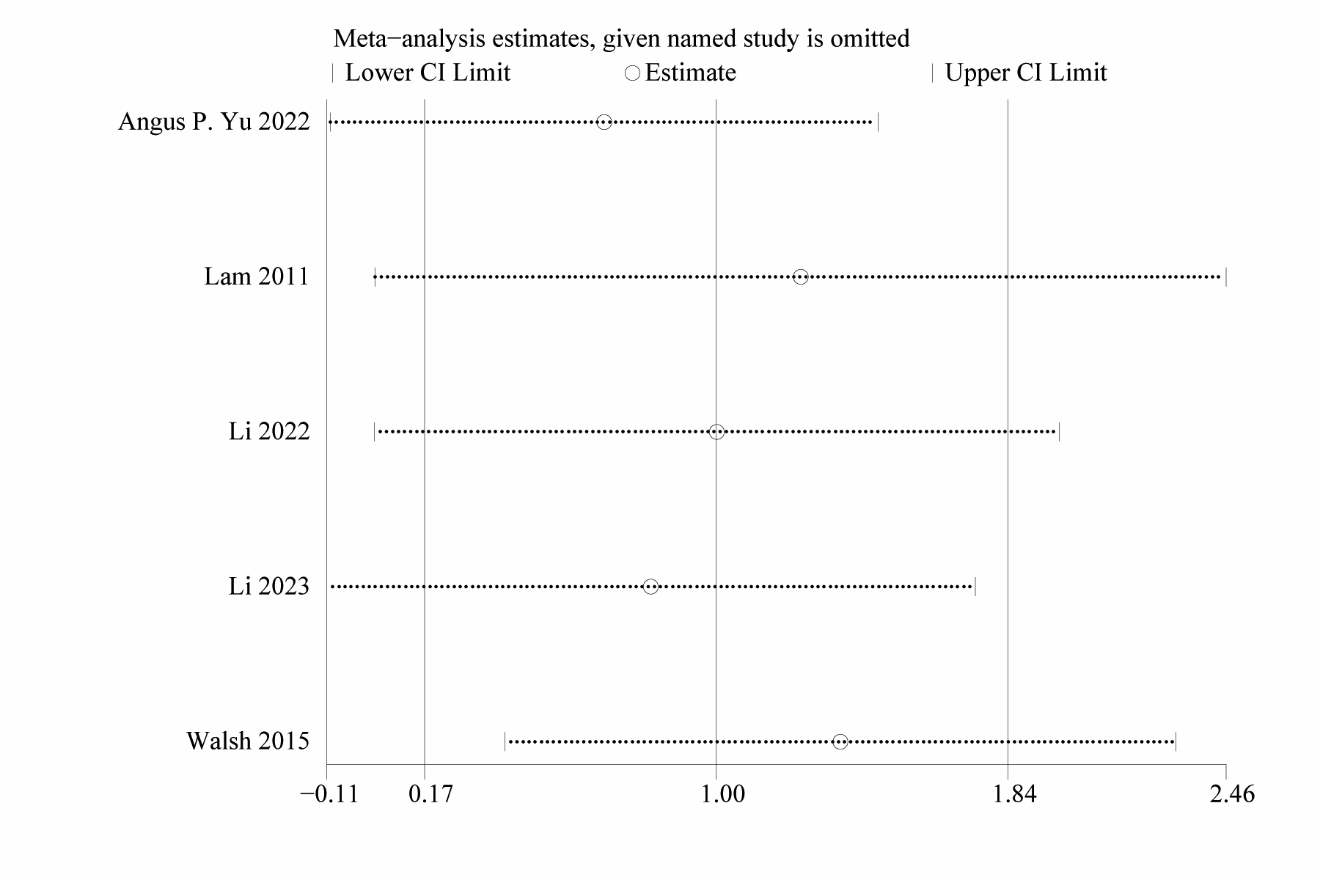


**Figure S8.** Sensitivity analyses for DS-F.


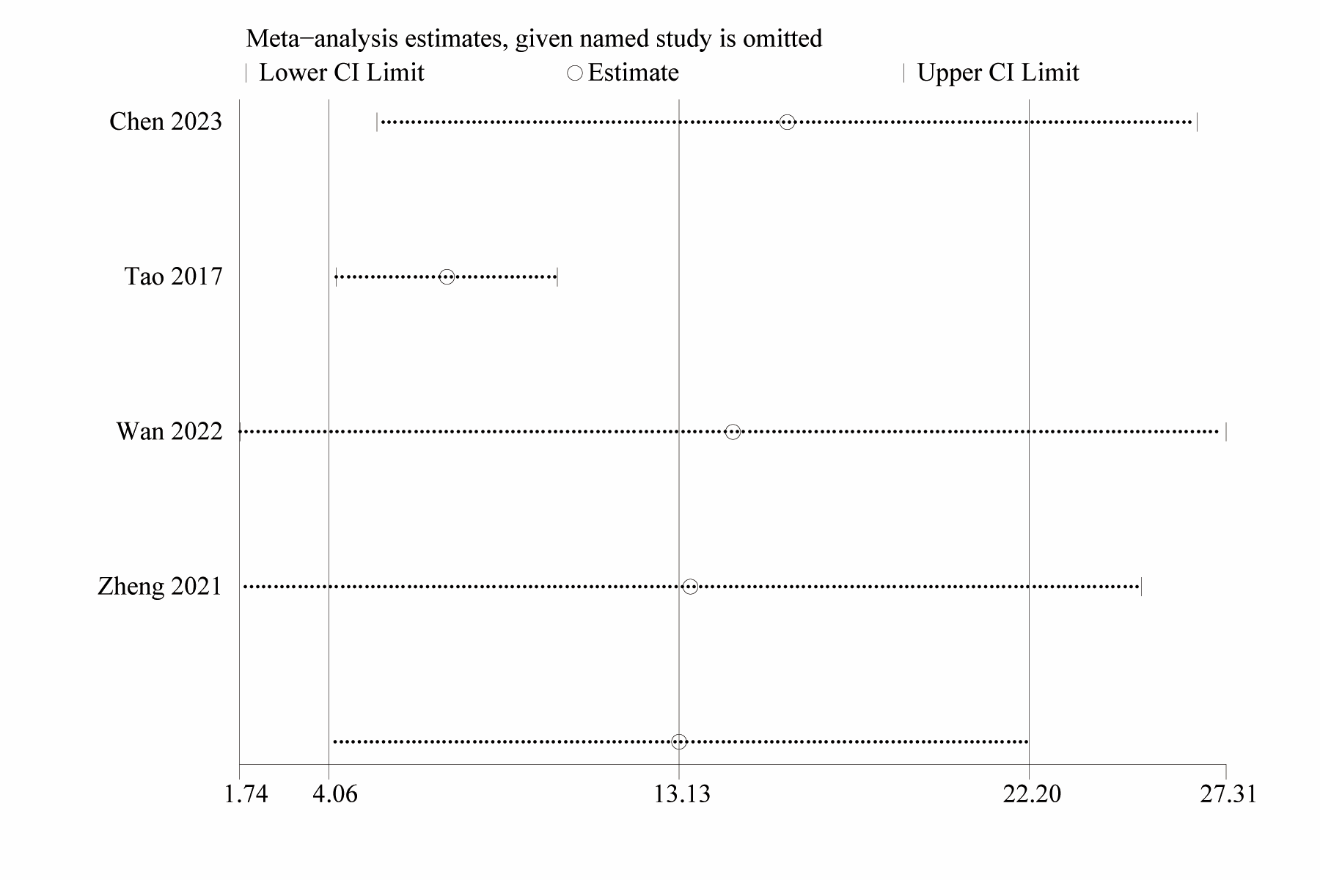


**Figure S9.** Sensitivity analyses for MQ.

A.


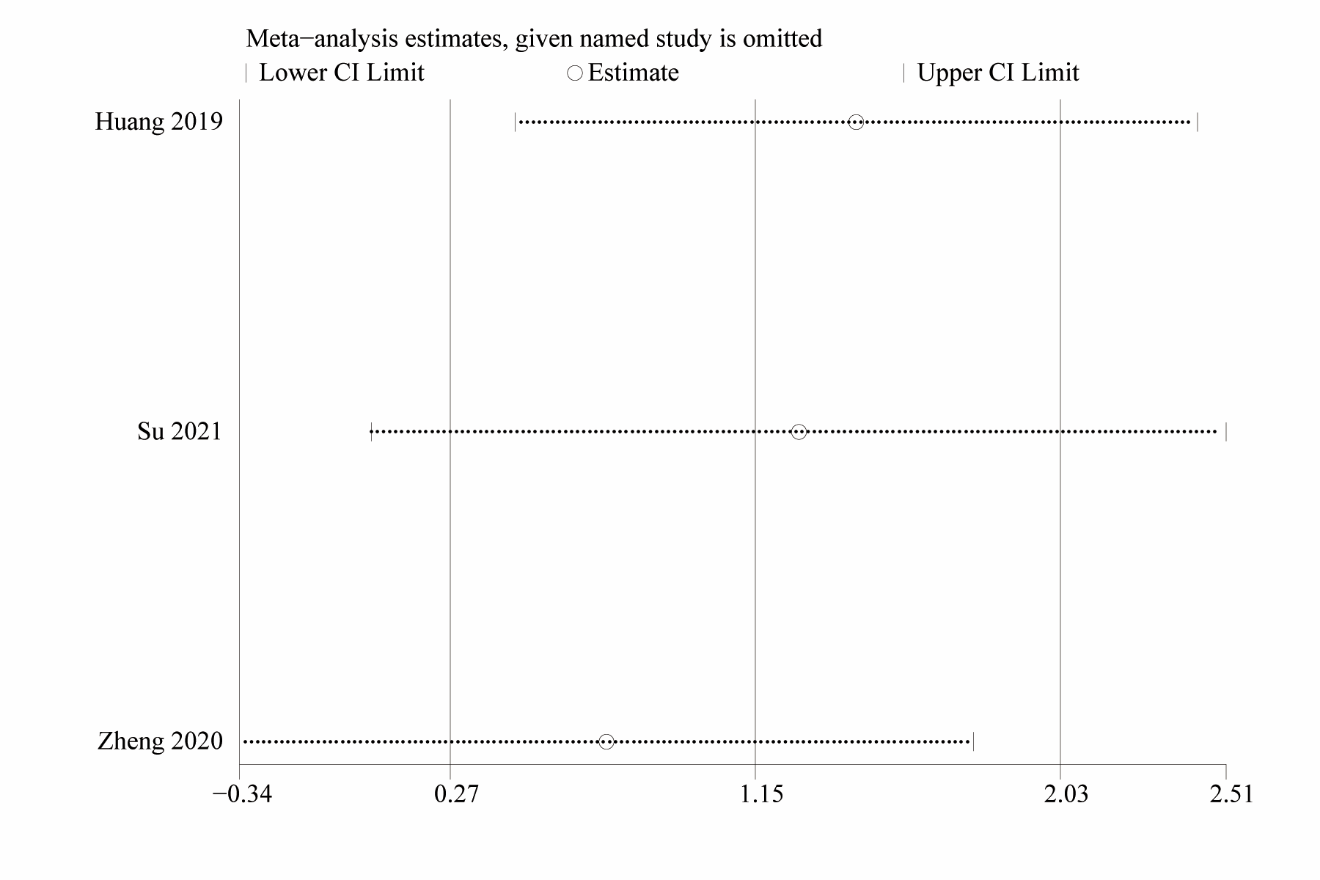


B.


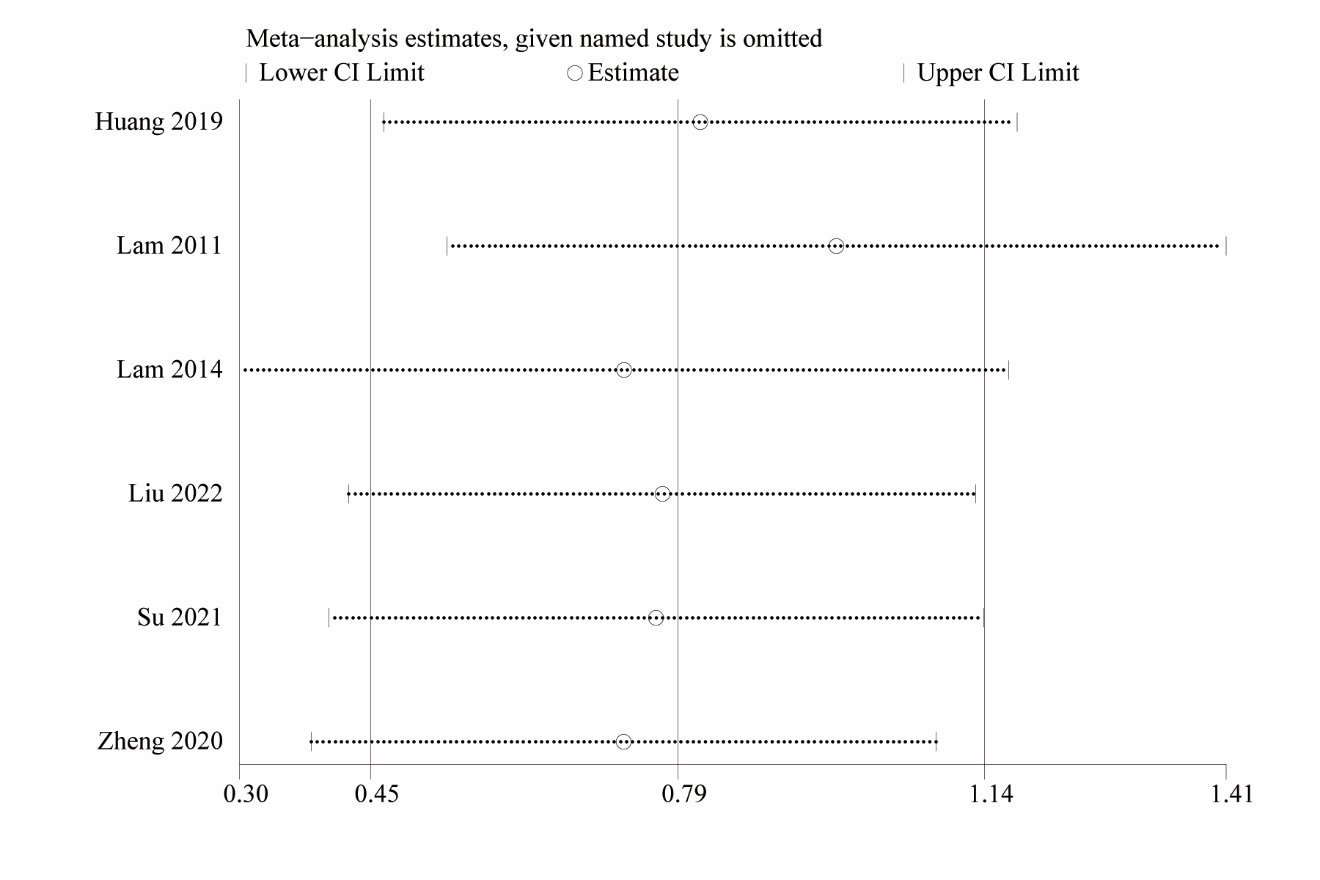


C.


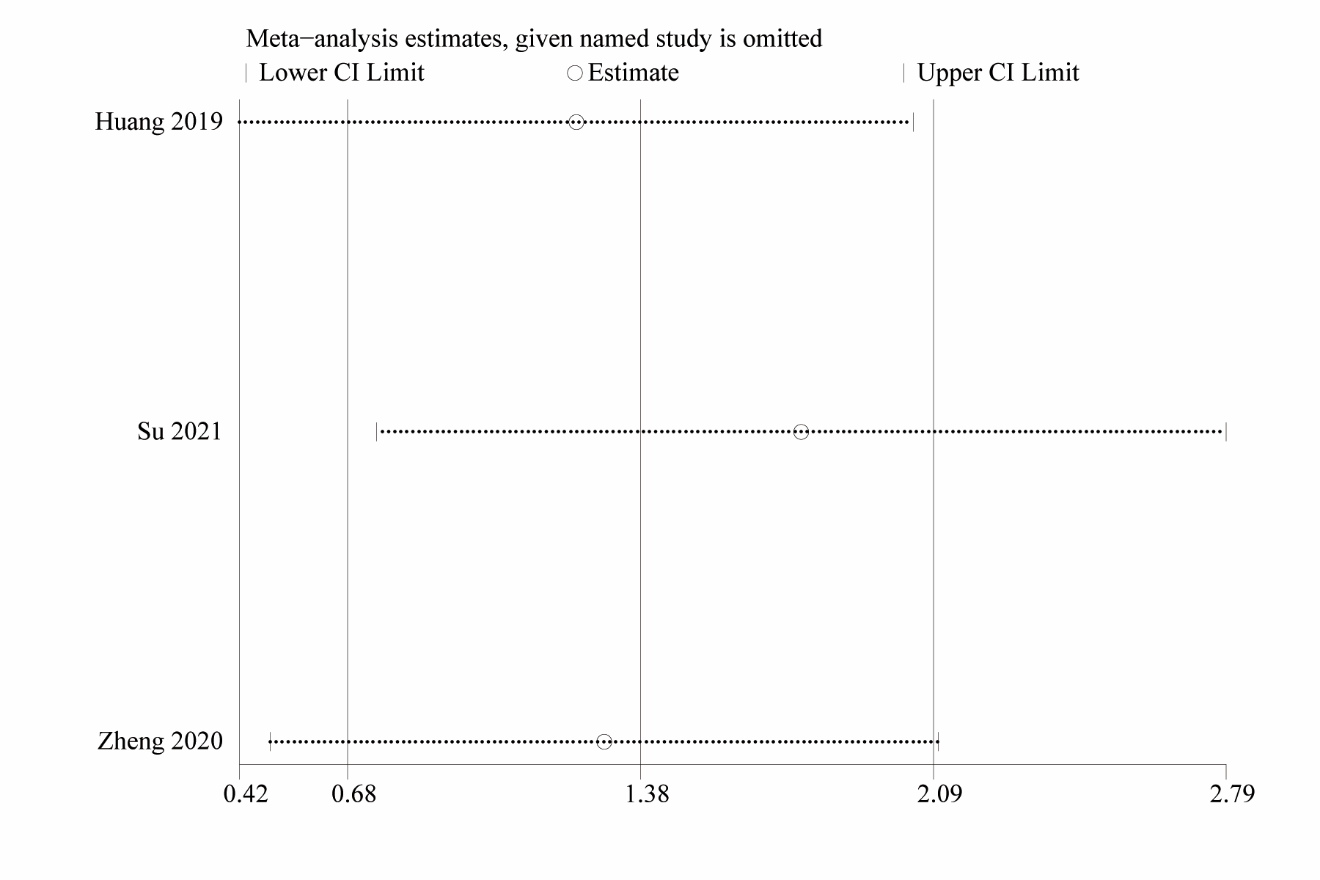


**Figure S10.** Sensitivity analyses for AVLT. A. immediate recall, B. short-term delayed, C. long-term delayed

**Appendix S6, Summary of univariate moderator analyses in MoCA**

Table S4

| Covariates | Coefficient | Standard error | 95%CI | P-value |
| --- | --- | --- | --- | --- |
| Location | -0.307 | 0.706 | [-1.861, 1.247] | 0.672 |
| Age | -0.251 | 0.542 | [-1.445, 0.942] | 0.930 |
| Gender | -0.351 | 0.712 | [-1.917, 1.215] | 0.632 |
| Health condition | -0.079 | 0.113 | [-0.327, 0.169] | 0.497 |
| Type of TCE | 1.468 | 0.381 | [0.629, 2.307] | 0.003 |
| Instruction mode | -0.357 | 0.821 | [-2.165, 1.451] | 0.672 |
| Characteristic of control | 0.268 | 0.565 | [-0.976, 1.513] | 0.645 |
| Duration of intervention | -0.317 | 0.156 | [-0.661, 0.026] | 0.066 |
| Session per week | 0.307 | 0.706 | [-1.246, 1.861] | 0.672 |
| Length per session | -0.220 | 0.294 | [-0.866, 0.427] | 0.470 |
| Note: beta coefficient in each cell obtained by fitting individual moderators into mixed-effects models respectively (n = 9). | | | | |

**Appendix S7, TCE-type subgroup analysis for the MoCA**


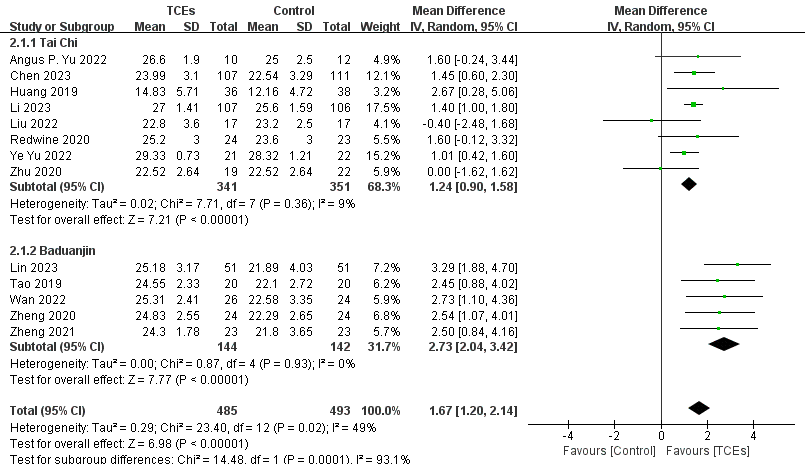


**Figure S11.** Forest plot of Montreal Cognitive Assessment (MoCA)

**Appendix S8, Funnel plot**


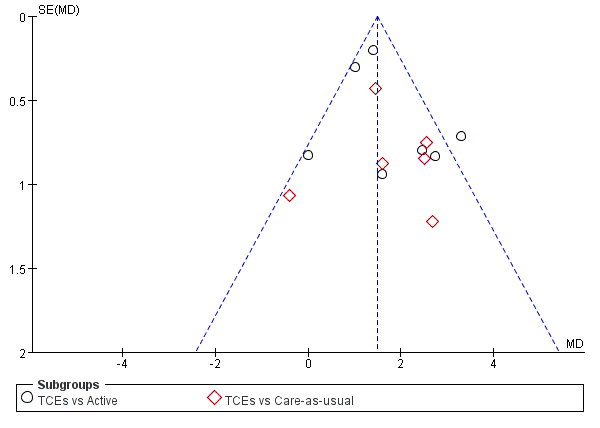


**Figure S12.** Funnel plot of Montreal Cognitive Assessment (MoCA).


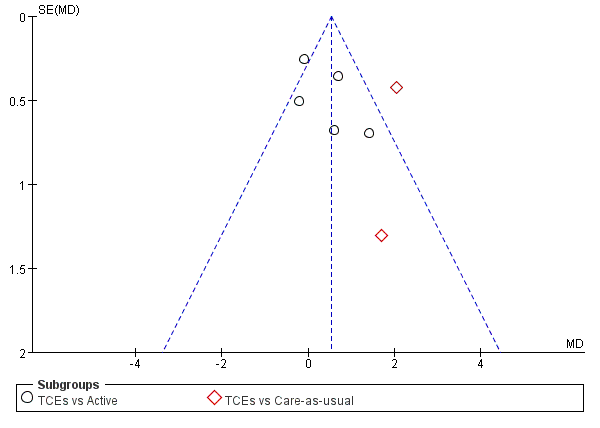


**Figure S13.** Funnel plot of Mini-Mental State Examination (MMSE).


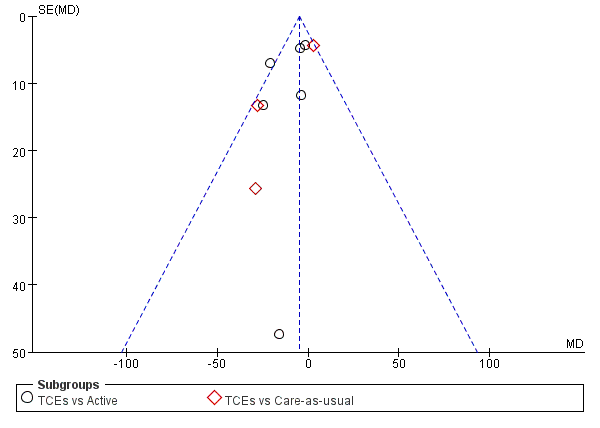


**Figure S14.** Funnel plot of Trail Making Test (TMT) B minus A (B-A)


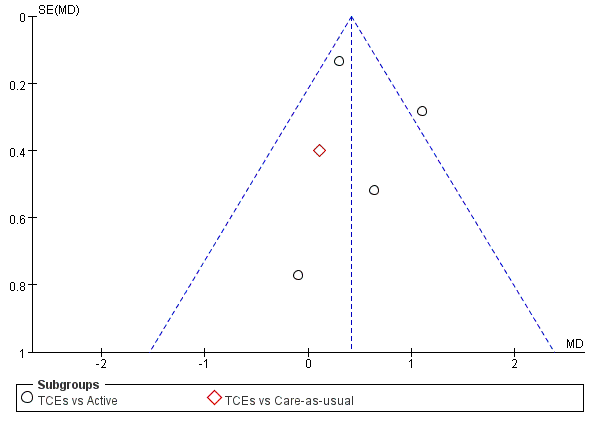


**Figure S15.** Funnel plot of Digit Span-Backwards (DS-B)

**Appendix S9, GRADE summary**

Table S5.

| **Outcome** | **No. of studies (participants)** | **Effect (MD, 95% CI)** | **Certainty of evidence (GRADE)** | **Downgrade reasons** |
| --- | --- | --- | --- | --- |
| **MoCA (primary)** | k = 13 (n = 978) | MD = 1.67 (1.20, 2.14) | **Moderate** | Risk of bias; inconsistency |
| MMSE | k = 7 (n = 793) | MD = 0.76 (0.04, 1.48) | **Low** | Risk of bias; imprecision |
| TMT (B-A) | k = 9 (n = 839) | MD = -7.96  (-15.34, -0.59) | **Low** | Risk of bias; inconsistency |
| Digit Span-Backward | k = 5 (n = 696) | MD = 0.48 (0.07, 0.90) | **Low** | Risk of bias; imprecision |

All included studies were randomized controlled trials; therefore, evidence started at high certainty.

Certainty was downgraded for risk of bias due to lack of allocation concealment and/or blinding in several trials.

Inconsistency was considered when moderate to substantial heterogeneity (I²) was observed.

Imprecision was considered when the total sample size was limited and/or confidence intervals were wide.

Publication bias was not downgraded due to limited power of funnel plots for several outcomes.
